# Orange Peel-Mediated Green Synthesis of ZnO and CuO Nanoparticles: Evaluation for Antimicrobial Activity and Biocompatibility in Tissue Engineering

**DOI:** 10.3390/ijms26188781

**Published:** 2025-09-09

**Authors:** Denisa-Maria Radulescu, Ionela Andreea Neacsu, Bogdan Stefan Vasile, Vasile-Adrian Surdu, Ovidiu-Cristian Oprea, Roxana-Doina Trusca, Cristina Chircov, Roxana Cristina Popescu, Cornelia-Ioana Ilie, Lia-Mara Ditu, Veronica Drumea, Ecaterina Andronescu

**Affiliations:** 1 Department of Science and Engineering of Oxide Materials and Nanomaterials, Faculty of Chemical Engineering and Biotechnologies, National University of Science and Technology POLITEHNICA Bucharest, 011061 Bucharest, Romania; 2 National Research Center for Micro and Nanomaterials, Faculty of Chemical Engineering and Biotechnologies, National University of Science and Technology POLITEHNICA Bucharest, 060042 Bucharest, Romania; 3 Romanian Academy of Scientists, 050045 Bucharest, Romania; 4 Research Center for Advanced Materials, Products and Processes, National University of Science and Technology POLITEHNICA Bucharest, 060042 Bucharest, Romania; 5 Department of Materials Science, Faculty of Materials Science and Engineering, Transilvania University of Brasov, 29 Eroilor Blvd., 500036 Brasov, Romania; 6 Department of Bioengineering and Biotechnology, Faculty of Medical Engineering, National University of Science and Technology POLITEHNICA Bucharest, Polizu 1-7, 011061 Bucharest, Romania; 7 Department of Life and Environmental Physics, National Institute for R&D in Physics and Nuclear Engineering Horia Hulubei, Reactorului 30, 077125 Magurele, Romania; 8 Department of Botany and Microbiology, Faculty of Biology, University of Bucharest, 060101 Bucharest, Romania; 9 S.C. BIOTEHNOS S.A., Gorunului 3-5, 075100 Otopeni, Romania

**Keywords:** green synthesis, nanoparticles, tissue engineering, copper oxide nanoparticles, zinc oxide nanoparticles, antimicrobial activity, biocompatibility, biofilm inhibition

## Abstract

The production of green nanomaterials has drawn considerable interest lately in the fields of tissue engineering and biomedicine. Thus, the environmentally friendly synthesis of ZnO and CuO nanoparticles (NPs) utilizing orange peel extract as a natural capping and reducing agent is the main focus of this study. Our comprehensive approach allows for a direct and systematic comparison of physicochemical attributes, biocompatibility, and antimicrobial activity under identical experimental circumstances, in contrast to other research that looked at individual nanoparticles under different conditions. The produced nanoparticles were characterized by techniques such as FTIR, XRD, SEM, TGA, and zeta potential assessment. MG-63 osteoblast-like cells, primary human dermal fibroblast BJ cells, and murine fibroblast L929 cells were used to evaluate biocompatibility using the MTT assay. The results showed dose-dependent cytotoxicity, especially above 25 µg/mL. Furthermore, both qualitative (growth inhibition zone diameter) and quantitative (minimum inhibitory concentration, MIC) techniques were used to assess the antimicrobial efficacy against *Candida albicans* and *Gram-positive* and *Gram-negative* bacteria. According to the obtained results, ZnO NPs showed broad-spectrum efficacy, whereas CuO NPs showed excellent antibacterial activity against *Gram-positive* bacteria (e.g., *S. aureus*, MIC = 0.313 μg/μL). The study highlights the potential of green-synthesized nanoparticles for utilization in biomedical applications, and it stresses the need for additional mechanistic research, including ROS measurement, to completely understand how they work.

## 1. Introduction

Thanks to technological developments and breakthroughs, nanotechnology has expanded quickly in recent years. The development of metallic and metal oxide nanoparticles (NPs) in the tissue engineering field has been particularly impacted by this increased attention, mostly because of their established antimicrobial properties [1,2]. Accordingly, nanotechnology makes it possible to create particles with specific sizes of less than 100 nm, which are subsequently used in a variety of industries, such as biomedicine, energy, the food industry, the environment, etc. [3,4]. Therefore, compared to bulk materials, NPs are regarded as materials with remarkable characteristics, such as particle distribution, shape, and an excellent surface-to-volume ratio because of their small size [5,6,7]. Furthermore, chemical, physical, or even biological methods can be used to create NPs in a wide variety of forms. Because of their improved antibacterial and antifungal properties, metal oxide NPs are being increasingly developed and studied, especially in the field of tissue engineering [8]. These nanomaterials include titanium oxide, silver, gold, zinc oxide (ZnO), copper oxide (CuO) nanoparticles, etc. [9,10,11,12,13,14].

The significant control over scaffold qualities, such as increasing the material’s mechanical strength and enabling regulated release of bioactive compounds, is another significant advantage of incorporating metal oxide NPs into tissue engineering [15]. Subsequent research has demonstrated that choosing the appropriate dosage, size, and size distribution minimizes the toxicity of the generated materials, despite other studies showing that metal nanoparticles were still detrimental [16]. The primary features of these materials, due to their nature and ease of functionalization, may result in an enhanced therapeutic impact [17]. Furthermore, because of their characteristics, which include high stability, an easy synthesis process, ease of manufacture in the selected morphologies, porosity, and size, and straightforward integration into hydrophobic and hydrophilic systems, metal oxide NPs can be used in tissue engineering to offer a variety of advantages [18].

The environmental impact of metal oxide nanoparticle production has continued to be the key concern in recent years. The most common synthesis routes are chemical and physical methods [19]. The synthesis processes indicated above frequently use hazardous chemicals that have the potential to cause environmental toxicity, carcinogenicity, or other problems. The main cause of these undesirable effects is the use of dangerous chemicals like stabilizers, reducing agents, and solvents. Additionally, using these hazardous solvents limits the range of biological or therapeutic applications for the created nanomaterials. As a result, a cleaner, more dependable, and environmentally friendly strategy is required [20]. Green synthesis pathways have drawn considerable attention in the research community in an effort to overcome these constraints. Green synthesis is currently the recommended synthesis pathway because it can use non-toxic (safer) solvents, prevent or minimize waste, and reduce derivatives and pollution [21]. Among the available natural sources, plants, fungi, bacteria, and algae are the most commonly used [22,23,24]. Among all metal oxide NP synthesis methods, using plant extracts and plant-derived materials is the most researched green synthesis route.

Although plant extracts have been used extensively for green synthesis, this study is the first to use a standardized protocol based on orange peel extract to synthesize two different metal oxide nanoparticles (NPs) (ZnO, CuO) under the same conditions. Their characteristics and biological performance can be carefully compared thanks to this method. According to thermal and spectroscopic investigations, the orange peel extract acts as a multipurpose agent during synthesis, aiding in precursor transformation and early-stage particle stabilization (Section 2.1, Section 2.2, Section 2.3 and Section 2.4). The extract’s function in forming intermediate phases and final NP crystallinity (Section 2.2) emphasizes its significance beyond waste valorization, even though calcination eliminates the majority of organic residues. Although energy-intensive calcination is still a barrier to sustainability, the method’s ease of use and utilization of inexpensive inputs (such as orange peel and metal nitrates) imply possible scaling.

Furthermore, our research integrates advanced characterization techniques (e.g., XRD, SEM, TEM, FTIR, and zeta potential analysis) with comprehensive biological assessments, including cytocompatibility, ROS generation potential, and antibacterial efficacy using MIC and MBEC assays. This comprehensive physicochemical and biological evaluation technique provides clear structure–function associations and useful data for NP design in wound healing and tissue engineering applications that require controlled dosage and minimal cytotoxicity. Our synthesis approach, which uses orange peel extract—a sustainable and underutilized waste product—not only adheres to green chemistry principles, but also improves the repeatability and scalability of NP production. This simultaneous focus on methodological standardization and multifunctional evaluation represents a considerable step forward from previous research, providing a more environmentally friendly alternative to nanoparticle synthesis with important biological applications in wound healing and tissue engineering.

Although plant-mediated green synthesis of metal oxide NPs is well-documented, this study provides a unique comparative perspective by employing a standardized orange peel extract-based synthesis protocol for three distinct metal oxides (ZnO and CuO NPs). This uniform methodology enables a direct and systematic evaluation of their structural, biological, and antimicrobial properties under identical conditions, addressing a critical gap in existing literature where comparative analyses are often lacking. Furthermore, our work integrates advanced characterization techniques (e.g., XRD, SEM, TEM, FTIR, zeta potential) with comprehensive biological assessments (cytocompatibility, ROS generation, MIC/MBEC assays), establishing clear structure–function relationships that are essential for tailoring NPs to specific biomedical applications.

## 2. Results and Discussion

### 2.1. Thermogravimetric Analysis Results

**Figure 1:**
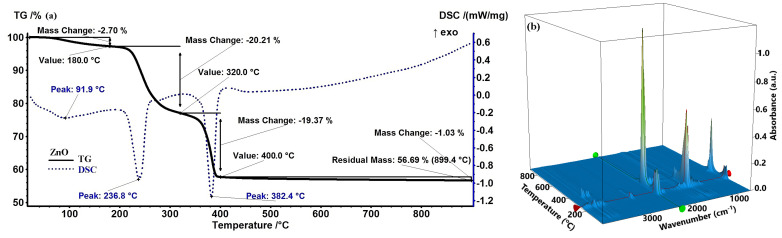
Thermogravimetric analysis of ZnO (**a**); FTIR 3D diagram of the evolved gases (**b**).

According to Figure 1, the ZnO precursor powder sample exhibits a 2.70% mass loss up to 200 °C, accompanied by a slightly endothermic thermal effect with a minimum of 91.9 °C. This process can be attributed to the loss of residual water molecules adsorbed on the particle surfaces. After 200 °C, the sample gradually and continuously lost around 20.21% of its mass, with an associated endothermic effect having a minimum at 236.8 °C. The FTIR 3D diagram of the evolved gases indicates the presence of peaks characteristic of water (ν_OH_ ~ 3400–3600 cm^−1^), carbon dioxide (double peak ~ 2316–2355 cm^−1^), and hydrocarbon fragments (ν_CHas_ 2919 cm^−1^) [25]. This, coupled with the endothermic effect, indicates that in the temperature interval 180–320 °C, the decomposition reactions of the organic part predominate, coupled with some partial oxidation reactions. Beyond 300 °C, the sample rapidly lost 19.37% of its mass up to 400 °C, with the associated strongly endothermic, and the sharp effect peaking at 382.4 °C. In this temperature interval, the FTIR 3D diagram indicates a lower quantity of hydrocarbon fragments, but a significantly higher amount of CO_2_ and CO, suggesting the decomposition of various organic acids and other organics with a C=O moiety. Furthermore, after 400 °C, the sample continued to slowly lose 1.03% of its mass up to 900 °C, mostly in a process of particle densification and elimination of the surface –OH moieties. The residual white mass at 900 °C represents 56.69%. This value is noticeably higher than the value of 27.36% corresponding to pure Zn(NO_3_)_2_·6H_2_O [26], or the theoretical value of 43.07% corresponding to anhydrous Zn(NO_3_)_2_. Additionally, the zinc nitrate final decomposition takes place between 280 and 300 °C, a temperature interval in which our sample exhibits no transformation [26]. All these results indicate that Zn(NO_3_)_2_·6H_2_O was transformed in the previous reaction with orange peel extract. Moreover, it can be concluded that the calcination temperature for the removal of the organic part from the ZnO sample is 400 °C. The possible phase changes observed in the DSC and TG curves will be further assessed by performing XRD analysis.

**Figure 2:**
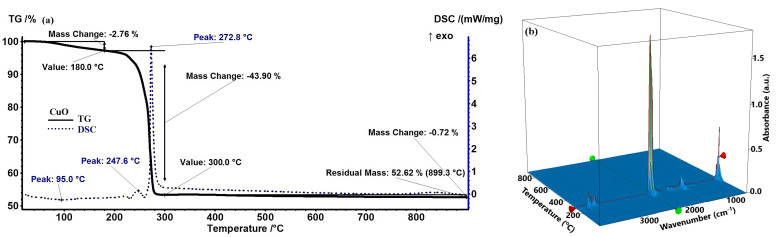
Thermogravimetric analysis of CuO (**a**); FTIR 3D diagram of the evolved gases (**b**).

For the CuO precursor powder (Figure 2), the sample shows a mass loss of 2.76% up to 190 °C, accompanied by a slight endothermic thermal effect peaking at 95 °C. This effect was attributed to the loss of surface-adsorbed water molecules. Beyond 200 °C, the sample rapidly loses 43.90% of its mass up to 300 °C, associated with a strong and sharp exothermic effect at 272.8 °C. Just at the start of this mass loss step, there are a few weak effects, the most important being the one that peaks at 247.6 °C. These effects suggest that the main reactions are the burning of organic material to generate the pure CuO phase. This conclusion is sustained by the FTIR 3D diagram of the evolved gases, which indicates CO_2_ as the main product of degradation, along with water and some traces of carbon monoxide. Additionally, some minute hydrocarbon fragments elimination can be observed just before the main spike of CO_2_.

After 300 °C, the sample continues to slowly lose 0.71% of its mass up to 900 °C, due to the elimination of surface –OH moieties. The residual black mass, at 900 °C, represents 52.62%. Also, in this case, the residual mass is significantly larger than the values for Cu(NO_3_)_2_·2.5H_2_O (34.39%) or Cu(NO_3_)_2_ (42.67%) [27]. As the non-calcined CuO sample also contains phytochemicals from orange peel extract, the high residual mass value can be considered an indicator of the successful reaction between Cu(NO_3_)_2_·2.5H_2_O and the organic extract. Additionally, the first decomposition step for copper nitrate is reported in literature to peak at 200 °C [27], while our sample is on a constant mass plateau at that temperature. These results suggest that a successful reaction between copper nitrate and orange peel extract took place. The experimental values indicate that the calcination temperature for the CuO sample is 300 °C.

### 2.2. X-Ray Diffraction (XRD) Analysis Results

**Figure 3:**
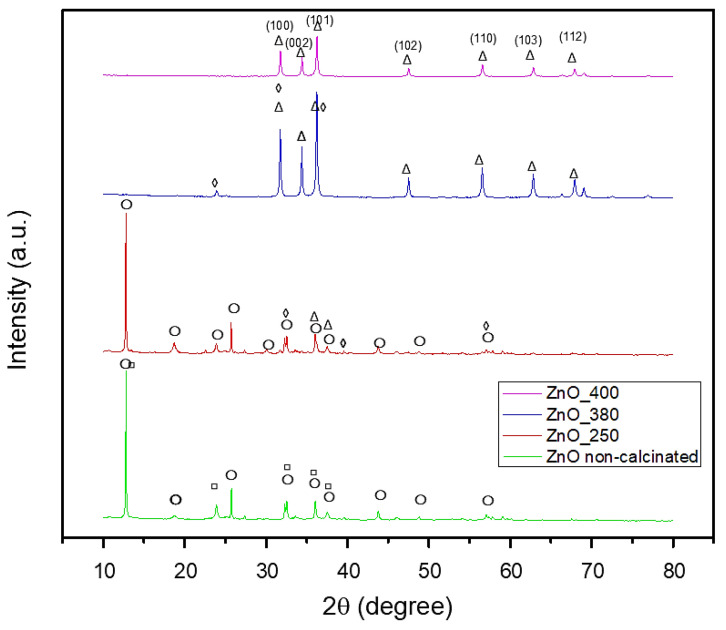
X-ray diffractogram performed on ZnO NPs, where **○**—Zn_3_(NO_3_)_2_(OH)_4_, ⸋—Zn(NO_3_)_2_·2Zn(OH)_2_, ◊—Zn(OH)2, and ∆—ZnO (green—untreated ZnO powder, red—calcinated ZnO NPs at 250 °C, blue—calcinated ZnO NPs at 380 °C, and purple—calcinated ZnO NPs at 400 °C).

**Figure 4:**
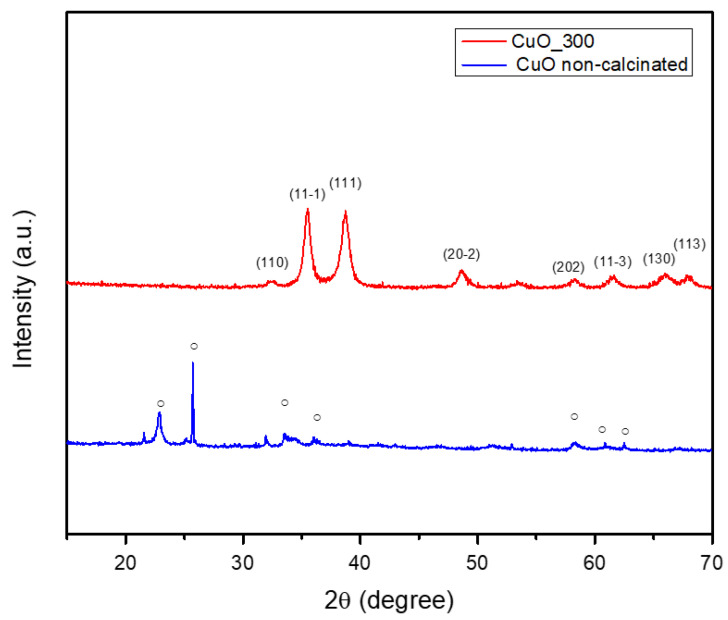
X-ray diffractogram performed on CuO NPs, where **○**—Cu_2_(OH)_3_NO_3_ (blue color—untreated CuO powder and red color—calcinated CuO NPs at 300 °C).

The crystalline phase composition of all green synthesized powders has been assessed by performing XRD analysis, with the results displayed in Figure 3 and Figure 4. For the non-calcinated ZnO-based powder (Figure 3), the synthesis exhibited two crystalline phases: Zn_3_(NO_3_)_2_(OH)_4_ (ICDD PDF4+ 04-014-0671) and Zn(NO_3_)_2_·2Zn(OH)_2_ (ICDD PDF4+ 00-018-1486). Incremental calcination of the ZnO-based powder at different temperatures demonstrated that the initial phases progressively decomposed, ultimately forming one unique crystalline phase of ZnO. By calcination at 250 °C the Zn(NO_3_)_2_·2Zn(OH)_2_ phase completely decomposed, leaving only Zn_3_(NO_3_)_2_(OH)_4_ and ZnO phases. The decomposition of the mentioned phase and the initial formation of ZnO could be associated with the endothermic effect exhibited in the TG curve. The ZnO phase, matching the PDF4+ 00-065-0725 file, displayed hexagonal symmetry. Elevating the calcination temperature to 380 °C enhanced the crystallinity, evidenced by the narrowing of the peak profiles and increase in their intensity, and, at the same time, led to the almost complete decomposition of the initial hydroxy nitrate phases, and thus the intensity of peaks associated with the intermediate compounds decreased until almost complete vanishing. This phenomenon is associated with the endothermic effect shown in Figure 1. However, a unique phenomenon occurs when the calcination temperature is increased to 400 °C, characteristic of ZnO phase formation. Although the ZnO phase is already formed, the intensity of ZnO diffraction peaks is slightly reduced. This effect is often attributed to the widely recognized behavior of larger ZnO crystallites at high temperatures. In this direction, it is known that larger ZnO crystallites tend to interpenetrate and form an interconnected intercrystalline network. This network can interfere with the long-range order and alignment of the crystallites, resulting in a small decrease in diffraction peak intensity despite the high degree of crystallinity. This phenomenon reflects the formation of a dense, interconnected ZnO matrix, which is crucial for certain applications where mechanical integrity or porosity is essential. By further elevating the heat treatment temperature to 400 °C, the ZnO phase was successfully synthesized, corresponding to the PDF4+ 01-090-0391 file, with hexagonal symmetry. The characteristic peaks were observed at 2θ = 31°, 34°, 36°, 47°, 56°, 63°, and 67°, and associated with the Miller indices (100), (002), (101), (102), (110), (103), and (112), respectively. The average crystallite size measured was 22.81 nm.

Figure 4 illustrates the successful synthesis of CuO crystalline phase using orange peel as a green synthesis route. The non-calcinated Cu-based powder displayed a single-phase composition of Cu_2_(OH)_3_NO_3_ (PDF4+ 96-901-2716) with orthorhombic symmetry. This phase was characterized by distinct peaks at 2θ = 12°, 22°, 25°, 32°, 34°, 40°, 53°, 58°, and 62°, corresponding to the Miller indices (002), (111), (112), (201), (121), (213), (216), (321), and (234), respectively. Following calcination at 300 °C, the CuO powder demonstrated the successful formation of the CuO phase, as indicated by the PDF4+ 96-901-4581 file. The obtained crystalline materials exhibited monoclinic symmetry. In this regard, the specific peaks were highlighted at 2θ = 32°, 35°, 38°, 48°, 58°, 61°, and 66°, corresponding to the Miller indices (110), (1$\bar{1}$1), (111), (2$\bar{0}$2), (202), (1$\bar{1}$3), and (310), respectively. Additionally, the average crystallite size of the CuO phase was determined to be 13.84 nm.

Hence, it could be summarized that the calcination process consistently facilitates the decomposition of hydroxide and nitrate groups, ultimately producing crystalline phases (ZnO and CuO). This transformation requires the elimination of volatile constituents (such as water and NOx) and the subsequent rearrangement of the remaining atoms into a stable oxide configuration. Understanding these phenomena is essential for customizing nanoparticle properties to fit specific applications, given that both crystalline structure and size possess a significant influence on the physical and chemical characteristics of the materials. Additionally, these results confirm oxide phase purity and initial composition but do not provide information on stability or dissolution kinetics in protein-containing media.

### 2.3. Scanning Electron Microscopy (SEM) Analysis Results

**Figure 5:**
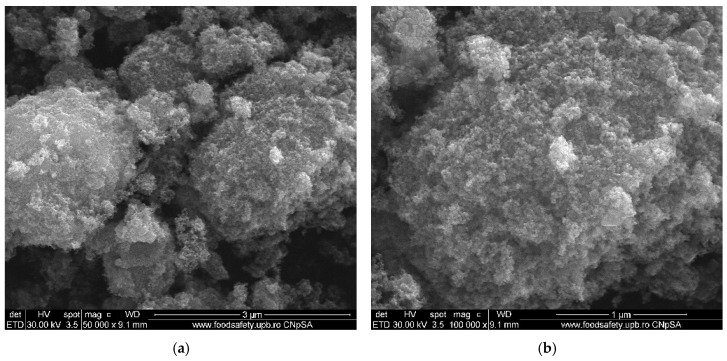

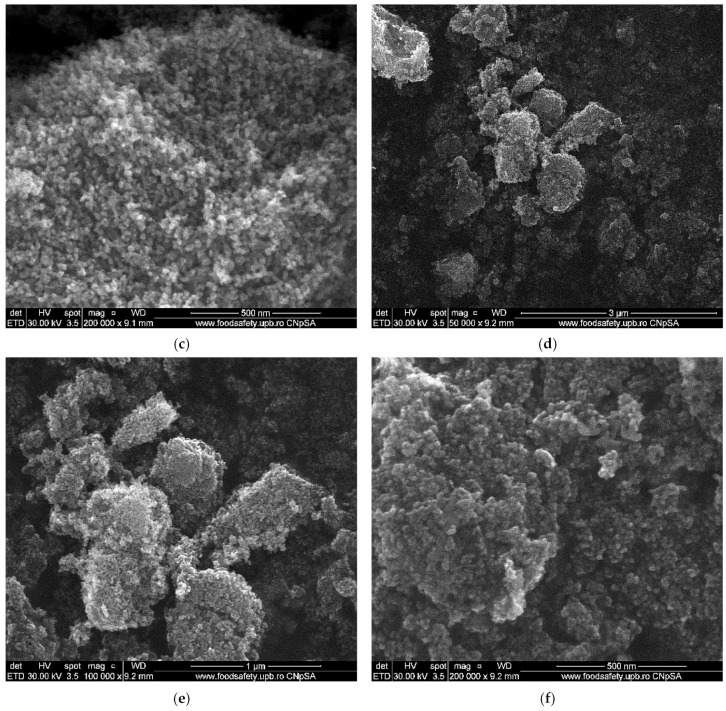
SEM images of green-synthesized ZnO NPs at (**a**) 50,000×, (**b**) 100,000×, and (**c**) 200,000× magnification, and of CuO NPs at (**d**) 50,000×, (**e**) 100,000×, and (**f**) 200,000× magnification.

The SEM micrographs presented in Figure 5 provide a detailed examination of the morphology and structure of the obtained samples. The performed analysis reveals that ZnO (Figure 5a–c) and CuO NPs (Figure 5d–f) possess a granular morphology, with a relatively uniform size distribution and a strong tendency to agglomerate. This tendency is primarily attributed to the large surface area of the NPs, which enhances their surface energy, leading to a natural inclination to cluster together. Also, the small size of the NPs, ranging from 3 to 20 nm, contributes to this agglomeration tendency. Thus, the high surface area and small particle size of the NPs result in increased surface energy and van der Waals forces, causing them to cluster together [28,29].

### 2.4. Fourier-Transform Infrared (FTIR) Spectroscopy Analysis Results

**Figure 6:**
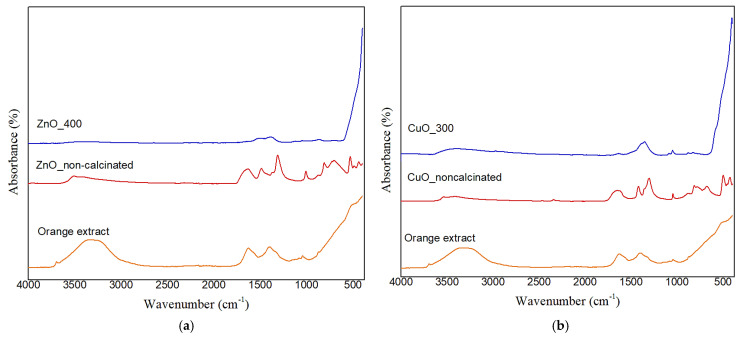
FTIR spectra of (**a**) ZnO NPs and (**b**) CuO NPs, where orange color—orange extract, red—untreated CuO and ZnO powder, and blue calcinated ZnO at 400 °C and calcinated CuO at 300 °C.

Figure 6 displays the FTIR spectrum of the synthesized metal oxide NPs. In this direction, strong absorption bands in the 400–600 cm^−1^ range are seen in all calcined samples (blue curves), and they have been assigned to metal–oxygen (M–O) stretching vibrations, confirming the production of ZnO and CuO NPs. These associations align with earlier findings for metal oxides produced using environmentally friendly methods [25]. As mentioned in the supplementary material of the Motelica et al. [30], crystal field effects and particular lattice vibrations associated with the wurtzite structure may be the source of the tiny peaks seen for ZnO in the 600–1000 cm^−1^ area. Adsorbed CO_2_ or leftover carboxylate groups (like citrate or acetate) that were not completely removed during calcination may be the cause of a faint doublet seen in some spectra at about 1500 cm^−1^. These vibrations have been detected in analogous green-synthesized materials and correlate to both symmetric and asymmetric stretching modes of COO^−^ groups. Traces of adsorbed carbon-containing compounds may still be present on the surface after calcination, even when organic materials are mostly eliminated.

Additionally, we observe that bands in the 1300–1600 cm^−1^ and 3400–3500 cm^−1^ regions in the non-calcined samples might be caused by a mix of the phytochemicals (flavonoids, terpenoids, and carboxylic acids) found in the orange peel extract, as well as adsorbed water, surface hydroxyl groups, or potentially undecomposed nitrates. These bands, however, are not present in the calcined samples, confirming the idea that heat treatment causes organics and nitrates to completely decompose. The results of XRD, which verify the lack of nitrate-related crystalline phases in the finished products, and TGA–FTIR evolved gas analysis, which failed to detect any NOₓ species, further support this interpretation. Therefore, these findings clearly show that the orange peel extract contributes chemically to the precursor transformation process rather than just serving as a physical medium.

The functional groups from the extract donate electrons that can react with metal ions, and the negative groups in the orange peel extract provide a stabilizing effect. Hence, it can be concluded that the interaction between the functional groups of the orange peel extract and the metal salts included electron donation from the extract to the inorganic species, facilitating their transformation into metallic oxide forms. During calcination, organic molecules decompose, purifying the material and leaving metal-oxide bonds. This process highlights the effectiveness of natural extracts in nanoparticle synthesis, offering both reactive groups and stabilizing agents for efficient and eco-friendly production [31].

In Section 2.1, TGA and FTIR of evolved gases provided clear evidence of the transformation of the metal nitrates in the presence of orange peel extract. Specifically, the thermal degradation profiles showed major weight losses corresponding to the decomposition of organic capping agents and the formation of inorganic residues. These residues, significantly higher than those expected from pure metal nitrate decomposition alone, indicate that preliminary interactions—most likely complexation—occurred between metal ions and organic compounds in the extract. Additionally, it appears that the metal ions had already reacted with the extract before calcination because the thermal profiles lacked the abrupt breakdown phases characteristic of unreacted metal salts.

The XRD investigation in Section 2.2, which identified crystalline intermediate phases such as hydroxy-nitrates (e.g., Zn_3_(NO_3_)_2_(OH)_4_ or Cu_2_(OH)_3_NO_3_), further supports this finding. These substances most likely originate when metal ions interact with the extract’s hydroxylated phytochemicals, and they break down when heated to produce metal oxide nanoparticles. But it is crucial to remember that XRD can only detect crystalline phases. FTIR and GC-MS data, which identify organic functional groups that contribute to the synthesis process, indirectly support the presence of any amorphous organic–inorganic intermediates that may exist before or during the early phases of calcination but are not detectable by XRD. The TGA–FTIR, XRD, and FTIR spectroscopy results provide a coherent picture of a multistep process: the formation of organo-metallic intermediates, followed by their thermally driven transformation into crystalline metal oxides. No evidence supports a reduction in metal ions to zero-valent metals in our system, in agreement with literature reports on green synthesis of metal oxide (not metallic) nanoparticles.

### 2.5. GC-MS Analysis Results

By performing the GC-MS assessment, it was concluded that the effectiveness of green synthesis utilizing orange peel extract results mostly from the synergistic activity of its several bioactive ingredients. Our orange peel extract’s GC-MS analysis verified a profile high in terpenoids, polymethoxyflavones, and fatty acids, which is in accordance with the sweet orange peels’ well-established phytochemical makeup [32,33,34,35]. The narrow size distribution and repeatable biological activities of our NPs indicate that, although a single cultivar would be necessary for complete uniformity, our use of a commercially available source yields an extract whose reducing and capping capacities are representative and should produce consistent results across batches.

The bioactive compounds found within the orange peel extract are terpenoids, polymethoxyflavones, fatty acids, amino acids, and sugar derivatives. These compounds interact in order to promote reduction, stabilization, and morphological control in the development of ZnO and CuO NPs, as summarized in Table 1. The terpenoids serve as both electron donors and metal ion complexing agents, resulting in regulated reduction and chelation. Related to the polymethoxyflavones, these compounds have a high redox potential and show dual-function behavior in reduction and chelation, whereas fatty acids operate as natural surfactants, facilitating particle capping, dispersion stability, and morphological modification. In addition, amino acids and sugar derivatives, particularly those transformed during extraction, help adjust particle shape and size.

The extraction and synthesis conditions optimize the coordinated biochemical activity. These procedures maintain and activate the biofunctional components, resulting in efficient and uniform nanoparticle production. This method explains the reproducibility and stability of the generated NPs.

Of particular interest is the orange peel extract’s chemical complexity, which suggests the existence of a multi-step, coordinated mechanism of NP production. Our GC-MS analysis showed a rich mixture of polymethoxyflavones (such as tangeretin and nobiletin), terpenoids, fatty acids, and derivatized sugars, each of which contributes independently to the reduction and stabilization process, in contrast to traditional green syntheses that depend on a single class of compounds (such as phenolics or flavonoids). Fatty acids help with particle capping and colloidal stabilization [36], amino acid derivatives modify morphology, and terpenoids and flavones decrease metal ions and encourage nucleation [37]. Therefore, it can only be summed up that these substances most likely work in tandem.

### 2.6. Zeta Potential Analysis Results

**Figure 7:**
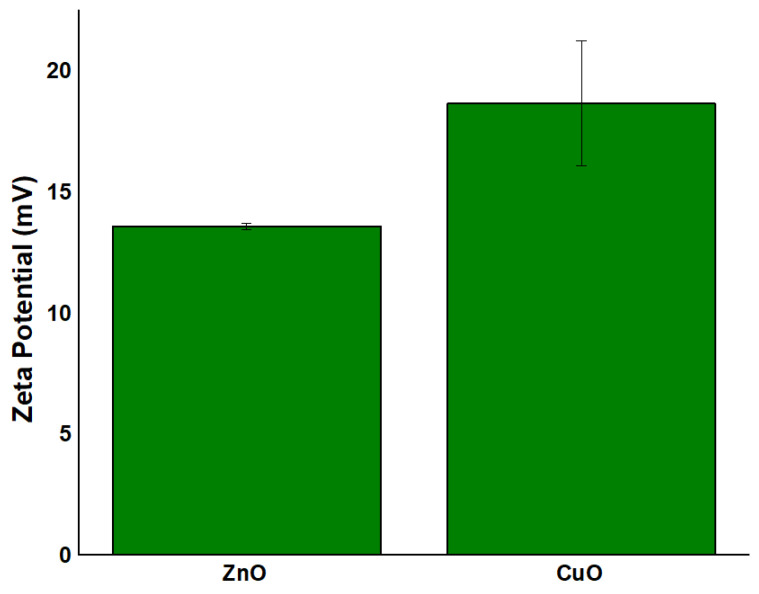
Zeta potential values for the pristine ZnO and CuO NPs measured in PBS 1× (expressed as mean ± SD, *n* = 3).

The zeta potential of the green-synthesized NPs was further assessed, as illustrated in Figure 7. Zeta potential is a key parameter that governs the interaction between NPs and bacterial cell surfaces. Its value plays a decisive role in determining the potential of the developed NPs to serve as drug delivery carriers. Typically, negatively charged carriers are preferred to enhance bacterial membrane permeability and promote antimicrobial activity [38]. Conversely, a higher positive surface charge on NPs can improve interactions with fungal cells through electrostatic attraction, facilitating the release of metallic ions and consequently inhibiting microbial growth [39]. As shown in Figure 7, all synthesized NPs exhibit positive zeta potential values, indicating a positively charged surface: 13.57 ± 0.57 mV for ZnO calcinated at 400 °C and 18.64 ± 2.58 mV for CuO calcinated at 300 °C. The moderate zeta potential values (ZnO: +13.57 mV; CuO: +18.64 mV) suggest limited colloidal stability, consistent with metal oxide NPs synthesized without additional stabilizers. While residual organic moieties (FTIR, Section 2.4) may contribute to surface charge, the extract’s primary role lies in precursor transformation rather than long-term stabilization. Although all NPs show positive surface charges, it is important to note that the magnitude of these values falls within the moderate range. Generally, zeta potential values between −30 mV and +30 mV are associated with moderate colloidal stability, while values exceeding these thresholds indicate high stability. The relatively low values observed (11.74–18.64 mV) may be insufficient to ensure strong electrostatic repulsion, potentially allowing for nanoparticle aggregation in suspension [40]. Interestingly, the trend observed in the zeta potential values (i.e., CuO > ZnO) is inverse to the electronegativity differences between each metal and oxygen: Zn-O (1.79) > Cu-O (1.54). As the covalent nature increases, the electron cloud of the oxygen anion becomes more distorted under the influence of the cationic electric field, which may lead to greater surface polarization and, consequently, a higher measured zeta potential. These findings underscore the intricate interplay between chemical bonding, nanoparticle surface properties, and the resulting electrokinetic behavior. They also emphasize the importance of using complementary structural and surface characterization methods to accurately understand nanoparticle performance and stability in biological environments. Additionally, it was concluded that the orange peel extract, while contributing to NP formation during synthesis, does not significantly improve long-term colloidal stability of the final calcined products, as the values remain in the moderate range.

### 2.7. Transmission Electron Microscopy (TEM) Analysis Results

**Figure 8:**
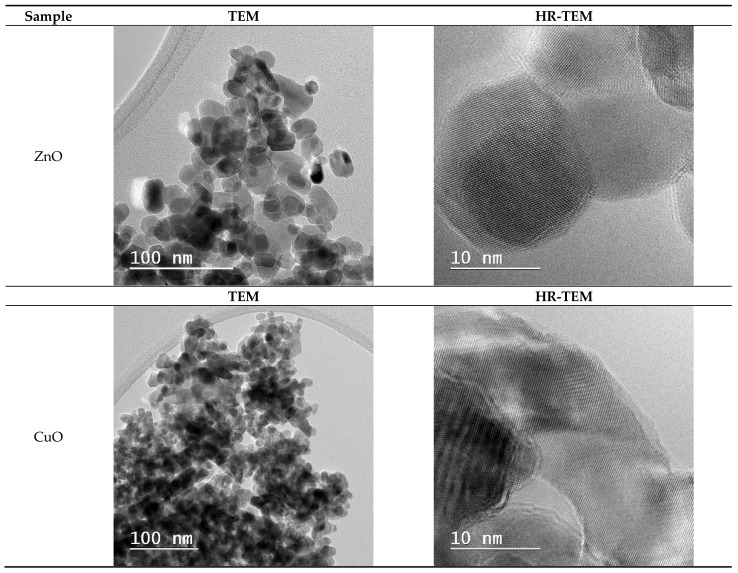
Transmission electron microscopy (TEM) images and high-resolution transmission electron microscopy (HR-TEM) images for ZnO and CuO samples.

Figure 8 shows TEM images of green manufactured metal oxide NPs. The CuO and ZnO samples have similar granular morphologies, as seen in SEM micrographs, with particle sizes ranging from 10 to 13 nm for CuO and around 20 nm for ZnO. Because of their large specific surface area and nanoscale size, these NPs tend to agglomerate due to insufficient electrostatic stabilization (as indicated by their low zeta potential values), rather than solely their small dimensions. The TEM observations are consistent with the SEM micrographs given earlier in the study, indicating that CuO and ZnO particles have an inherent tendency to cluster together due to their high surface energy, a feature of nanoscale materials with large specific surface areas. This correlation between TEM and SEM provides a strong basis for conclusions regarding particle size and morphology. HRTEM images reveal that all NPs are crystalline, with ZnO and CuO exhibiting well-defined lattice fringes.

**Figure 9:**
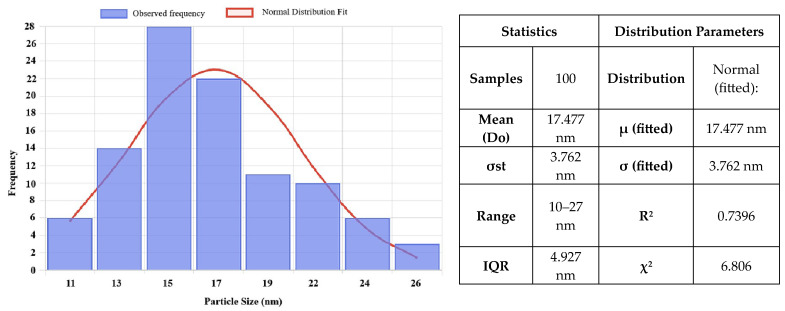
Histogram of ZnO NP sizes with descriptive statistics and fitted distribution parameters. The sample (*n* = 100) shows a mean size of 12.586 nm, low variability (CV = 10.0%), and strong normality (R^2^ = 0.942), indicating uniform synthesis.

**Figure 10:**
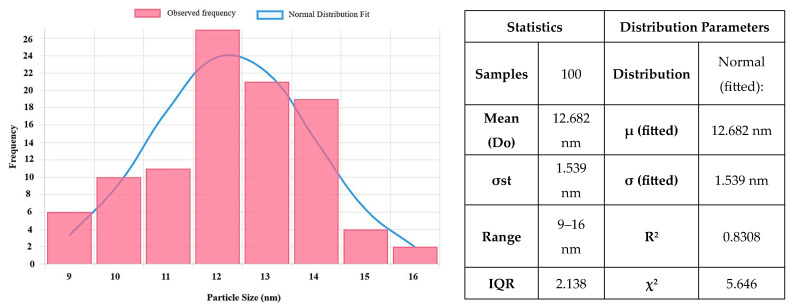
Histogram of CuO NP sizes with descriptive statistics and fitted distribution parameters. The sample (*n* = 100) shows a mean size of 12.682 nm, low variability (CV = 12.1%), and strong normality (R^2^ = 0.8308), indicating uniform synthesis.

Figure 9 and Figure 10 describe the size distribution profiles of all green-synthesized NPs. Related to ZnO NPs, they have demonstrated the largest mean size of 17.47 ± 3.76 nm. However, their distribution is remarkably uniform, with particle sizes ranging from approximately 10 to 27.5 nm. For instance, the majority of ZnO NPs can be seen at sizes between 15 and 18 nm. This constant size distribution demonstrates the effectiveness of the green synthesis process using orange peel extract in creating NPs with well-defined dimensions. Such consistency is especially useful for improving the stability, dispersibility, and interaction of NPs with biological systems, all of which are critical in biomedical applications. Moreover, the reduced particle sizes and uniform distribution contribute to an increased specific surface area, which is critical for improving surface reactivity, enhancing bioavailability, and boosting the overall biological performance of the NPs.

Furthermore, CuO NPs (Figure 10) have a slightly lower mean size of 12.682 ± 1.24 nm, with a narrow distribution between 10 and 16 nm. This uniform size range reflects effective control over particle growth during the synthesis process, which is advantageous for achieving stability and reproducibility. The comparison between particle sizes measured by SEM, TEM (Figure 5 and Figure 8), and crystallite sizes calculated from XRD (Figure 3 and Figure 4) reveals key insights into the crystallinity of CuO and ZnO NPs. For CuO NPs, the SEM sizes range from 10 to 20 nm, with an average of 12.65 ± 1.53 nm, which closely aligns with the crystallite size of 13.84 nm obtained from XRD. This close match suggests that CuO NPs are likely monocrystalline, meaning each particle consists of a single crystallite. For ZnO NPs, the SEM size range is 3–20 nm, with an average particle size of 17.47 ± 3.76 nm, while the crystallite size determined from XRD is slightly larger, at 22.81 nm. The differences are most probably due to the method reporting different sizes of the samples. The crystallite size is an average calculated for the whole sample, while particle size is determined as an average for a limited number of structures from SEM. The closer values for particles and crystallite size suggest that each nanoparticle observed in SEM consists of single crystallites, confirming ZnO NPs are likely monocrystalline or contain few crystallites per particle.

### 2.8. Cell Viability Assessment Results

**Figure 11:**
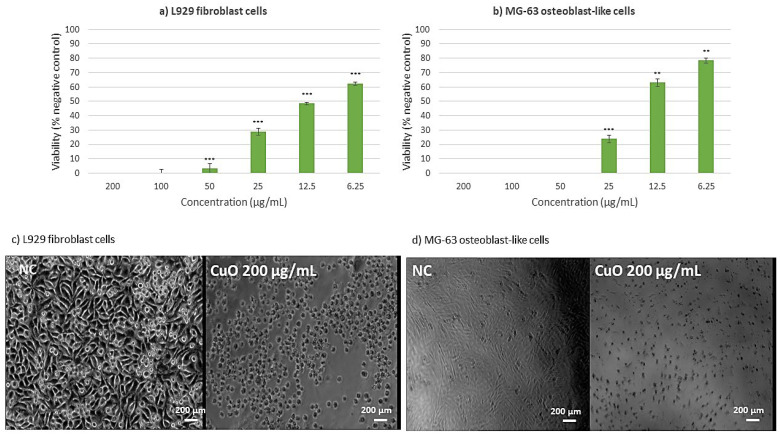
Cytotoxicity assessment of green-synthesized CuO NPs after 4 days of incubation for (**a**,**c**) murine fibroblast L929 cells, and (**b**,**d**) MG-63 osteoblast-like cells. The negative control consisted of cells incubated only with complete culture medium. Data are expressed as mean ± SD, where *n* = 3. Statistical analysis was performed using Student’s *t*-test (** *p* ≤ 0.01, *** *p* ≤ 0.001).

**Figure 12:**
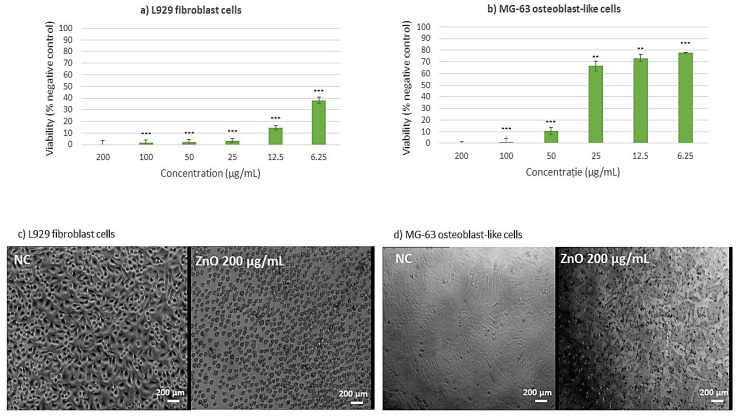
Cytotoxicity assessment of green-synthesized ZnO NPs after 4 days of incubation for (**a**,**c**) murine fibroblast L929 cells, and (**b**,**d**) MG-63 osteoblast-like cells. The negative control consisted of cells incubated only with complete culture medium. Data are expressed as mean ± SD, where *n* = 3. Statistical analysis was performed using Student’s *t*-test (** *p* ≤ 0.01, *** *p* ≤ 0.001).

To investigate the cytotoxic effects across multiple cell lines, an MTT assay was performed, and presented in Figure 11 and Figure 12. The investigation of CuO NPs (Figure 11) revealed significant cytotoxic effects across multiple cell lines. In L929 murine fibroblast cells (Figure 11a), exposure to CuO NPs at concentrations exceeding 25 µg/mL resulted in substantial cytotoxicity. At the same time, at the lower concentration of 6.25 µg/mL, cellular viability was reduced by 40% compared to the negative control, indicating moderate cytotoxic effects even at reduced concentrations. The cytotoxicity of CuO NPs on MG-63 osteoblast-like cells (Figure 11b) was clearly dose-dependent. Furthermore, morphological analyses indicated considerable alterations in cellular structure and widespread cell death, implying that CuO NPs may cause both metabolic disturbance and structural damage to cells. This comprehensive cytotoxic effect indicates that CuO NPs may act through multiple cellular damage pathways.

Regarding ZnO NPs (Figure 12), the cytotoxicity assessment was conducted to evaluate cellular metabolic activity. Studies on L929 murine fibroblast cells revealed that ZnO NPs exhibited considerable dose-dependent cytotoxicity at concentrations higher than 25 µg/mL, indicating severe cellular stress and metabolic dysfunction (Figure 12a). At a lower dose of 6.25 µg/mL, a small cytotoxic reaction was found, with a 40% reduction in cellular viability compared to the negative control, indicating a threshold impact in the cytotoxic response. The cytotoxic profile of MG-63 osteoblast-like cells (Figure 12b) revealed a unique pattern: higher concentrations (50–200 µg/mL) resulted in considerable cytotoxicity, whereas lower concentrations (6.25–25 µg/mL) maintained cellular viability within biocompatible ranges. This differential reaction shows that ZnO NPs are sensitive to specific cell types. This result shows that specific cell types have different sensitivity to the ZnO NPs [41].

This comprehensive analysis suggests that among the tested metallic oxide NPs, both ZnO and CuO exhibit varying degrees of cytotoxicity depending on concentration and cell type. These findings have significant implications for the potential biomedical applications of these NPs, particularly in contexts where cellular interaction is crucial. While contact-mediated effects, ROS generation, and possible ion release may all contribute to the observed outcomes, the present study did not directly measure dissolution or ionic leaching. These mechanisms should therefore be regarded as hypotheses requiring further validation.

### 2.9. Qualitative Evaluation of Antimicrobial Activity Assessment

**Figure 13:**
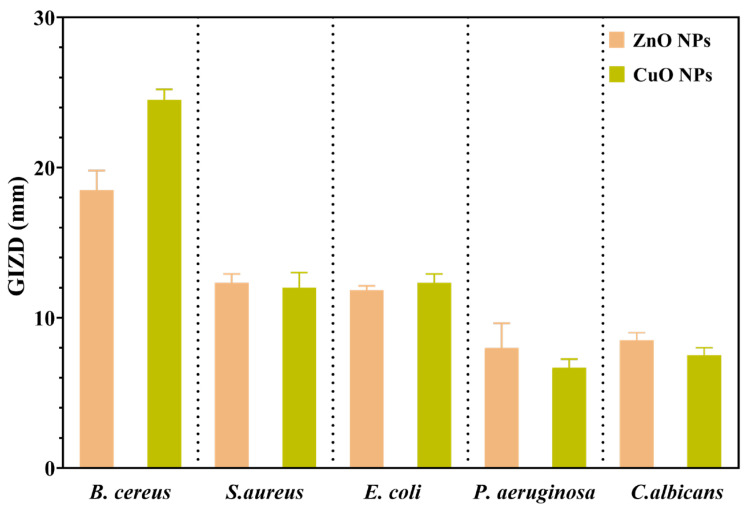
GIZD values of metal oxide NPs. Values are expressed as mean ± standard deviation (SD) in millimetres (mm). Each measurement was performed in triplicate (*n* = 3).

Firstly, the antimicrobial properties of metal oxide NPs were qualitatively evaluated by measuring the growth inhibition zone diameters (GIZDs) that appeared near the spot and expressing them as mean values ± SD (Figure 13).

The antimicrobial profiles of each NP studied are displayed in Figure 13. The highest GIZDs were observed in the case of *Gram-positive* bacteria, followed by *Gram-negative* bacteria and *C. albicans*. Furthermore, both ZnO and CuO NPs generated the greatest sensitivity of all strains tested in this study. In this direction, ZnO and CuO NPs are very effective at producing reactive oxygen species (ROS) such as hydroxyl radicals and superoxide anions, which cause oxidative stress in microbial cells [42]. In the case of ZnO NPs, *B. cereus* was found to be the most susceptible strain, while *P. aeruginosa* is the most resistant, similar to the results reported in [43]. This oxidative damage harms critical cellular components such as lipids, proteins, and DNA, affecting membrane integrity and resulting in cell death.

### 2.10. Quantitative Evaluation of Antimicrobial Activity Assessment

**Figure 14:**
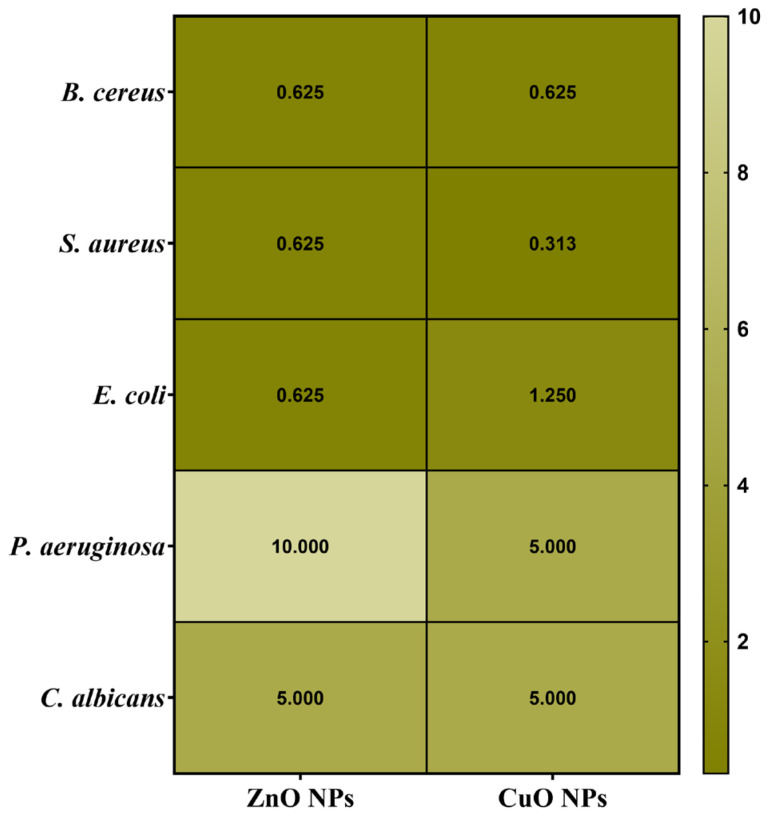
Heat map of MIC values. The scale bar shows the variations in the sensitivity of tested strains from the highest (green) to the lowest (light green).

Secondly, the antimicrobial assessments were continued with quantitative evaluation by determining the minimum inhibitory concentration (MIC) values (Figure 14). Data results marked in dark green suggest the significantly lowest MIC values and highest sensitivity of strains.

Understanding the efficiency of these NPs starts from understanding that lower MIC values imply more antimicrobial potency because they represent the smallest amount of material required to suppress bacterial growth. Notably, the MIC assessment supported the previously obtained qualitative results by quantitatively demonstrating the antimicrobial properties of the NPs, as shown in Figure 14. *S. aureus* showed high sensitivity to CuO NPs, inhibiting growth at a concentration in the range of 0.313 μg/μL. Previous research has shown that smaller NPs can effectively penetrate bacterial cell membranes, resulting in strong inhibitory effects against *B. cereus* and *E. coli* (MIC values ranging from 0.310 to 2.500 μg/μL) [44]. Otherwise, CuO NPs determined the highest sensitivity on *S. aureus* (0.313 μg/μL), followed by *B. cereus* (0.625 μg/μL) and *E. coli* (1.250 μg/μL). Additionally, their impact becomes notably reduced against *P. aeruginosa* and *C. albicans*, requiring a higher concentration of 5.000 μg/μL to achieve inhibition.

On the other hand, ZnO NPs have a distinct pattern of efficacy. They exhibit substantial antibacterial activity against *B. cereus*, *S. aureus*, and *E. coli*, with MIC values of 0.625 μg/μL. However, their efficiency is reduced dramatically against *P. aeruginosa*, necessitating a greater dosage of 10.000 μg/μL. They show moderate activity against *C. albicans*, with an MIC of 5.000 μg/μL. This research reveals several larger tendencies. Firstly, *P. aeruginosa* and *C. albicans* consistently demonstrate moderate sensitivity to all NP types, requiring larger concentrations for effective inhibition. In contrast, *Gram-positive* bacteria, specifically *B. cereus* and *S. aureus*, generally exhibit higher sensitivity to these metal oxide NPs. This differential effectiveness across various strains highlights how the size, morphology, and specific properties of NPs influence their antimicrobial activity, with smaller particles generally showing enhanced ability to penetrate cell membranes and exert their effects, as consistently demonstrated throughout the literature [44].

### 2.11. Semiquantitative Assessment of Microbial Adherence to the Inert Substratum Results

**Figure 15:**
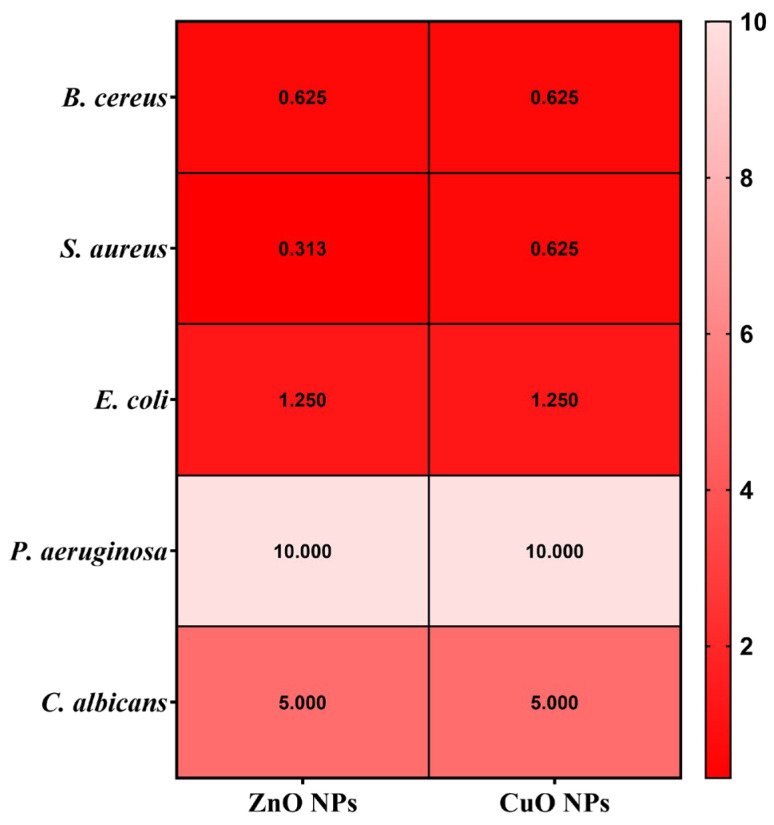
Heat map of MBEC values. Data results marked in red suggest the significantly lowest MBEC values. The scale bar shows the variations in the sensitivity of tested strains from the highest (red) to the lowest (pink).

The minimal biofilm eradication concentration (MBEC) assessment gives critical information about how well these NPs can inhibit the adherence of biofilms. The antimicrobial effects of the obtained NPs were evaluated by assessing their effect on pathogenic strain adhesion to an inert substrate. The MBEC values are illustrated in Figure 15 and confirm the qualitative and quantitative results (Figure 13 and Figure 14).

Several notable trends could be observed when looking at the specific patterns in the MBEC values. In this direction, for *B. cereus*, ZnO and CuO NPs demonstrate better effectiveness with lower MBEC values of 0.625 μg/mL. This indicates that ZnO and CuO NPs are especially effective at penetrating and destroying *B. cereus* biofilms. *S. aureus* exhibits varying sensitivities, with ZnO NPs accomplishing the most effective biofilm eradication at 0.313 μg/mL, while CuO NPs require a higher concentration of 0.625 μg/mL. This pattern is consistent with the general tendency of some NPs to have increased efficacy against *Gram-positive* bacteria. *E. coli*, a *Gram-negative* bacterium, showed considerable resistance to biofilm removal and required larger doses of all NP types (1.250 μg/mL for ZnO and CuO NPs). In addition, *P. aeruginosa* exhibited the highest resistance to ZnO and CuO (MBEC = 10.000 μg/mL). Related to the fungal species *C. albicans*, this strain has displayed variable sensitivity, with ZnO and CuO requiring concentrations of 5.000 μg/μL.

## 3. Discussion

Even though there is ample evidence of the environmentally friendly production of metal oxide nanoparticles (NPs) by plant extracts, our study is focused on the consistent application of orange peel extract to the synthesis of ZnO and CuO NPs under the same conditions. The orange peel extract’s phytochemicals may have a variety of roles, such as reducing, chelating, stabilizing, and morphology-directing agents during NP formation, according to extensive physicochemical characterization (TGA/DSC, XRD, FTIR, GC-MS, SEM/TEM, and zeta potential). In particular, thermal investigation shows how these bioactive chemicals alter precursor salts, suggesting a chemical interaction that goes beyond simple nitrate decomposition. Most organic residues are eliminated by the calcination process that follows, producing extremely crystalline oxide phases.

As previously mentioned, our study enables a direct comparison of ZnO and CuO NPs under the same experimental circumstances, in contrast to earlier research that concentrates on individual nanoparticles. This comparative perspective offers a path for improving the design of NPs for specific biomedical applications by bringing insights into how minor changes in crystallinity, size, and surface charge affect biocompatibility and antibacterial efficacy. Our approach allows for direct, side-by-side comparison of physicochemical properties and biological performances, in contrast to previous works that concentrated on individual nanoparticles synthesized under various conditions. This reveals crucial structure–function relationships necessary for tissue engineering and antimicrobial applications. This methodological consistency addresses a critical gap identified by Thi et al. [31], Ali et al. [45], and many other studies, which emphasized the need for standardized protocols in green nanoparticle synthesis.

In addition, the TGA assessment revealed that the initial weight loss detected below 200 °C from all samples is attributed mostly to the elimination of adsorbed moisture on the particle surfaces, where residual moisture and organic components from the plant extract are common [46]. The second significant weight loss, between 200 and 400 °C, corresponds to the breakdown of the capping phytochemicals that are to be found in the uncured samples. The endothermic and exothermic peaks in this range correspond to decomposition and oxidation, respectively, of the organic residues. Additionally, the residual mass of all samples is significantly higher than the theoretical value of the metallic nitrates. As all samples also contain phytochemicals, we can conclude that indeed the metal nitrates were transformed in the reaction with the orange peel extract. Moreover, the presence of such separate thermal processes is a sign of successful green synthesis, as these changes confirm the stepwise oxidation of metal precursors and their complete conversion to oxide forms at higher temperatures [47]. This phase evolution is critical for producing stable, crystalline NPs appropriate for biological applications.

Moreover, XRD investigation confirmed the obtained NPs. We found that calcination also produced phase-pure crystalline CuO, ZnO, and MgO with reduced agglomeration, which is in line with our previous work on green-synthesized nanoparticles [48]. While Doan Thi et al. [31] achieved ZnO NPs with sizes of 25–35 nm using orange peel extract, our optimized protocol produced smaller, more narrowly distributed ZnO NPs (17.47 ± 3.76 nm). Similarly, the CuO NPs (12.65 ± 1.24 nm) are significantly smaller than those reported by Murugan et al. [13] using various plant extracts (20–50 nm). This size reduction is attributed to our systematic optimization of extraction conditions and metal salt concentrations. For green-synthesized metal oxides, the crystallographic investigation reveals significant structure–property connections that have not been documented before. As reported in the literature, our ZnO NPs show the expected hexagonal wurtzite structure (space group P63mc), but they have significantly higher crystallinity, as shown by their distinct, sharp peaks. The crystallite diameters (22.81 nm for ZnO and 13.84 nm for CuO) exhibit an inverse relationship with changes in electronegativity, which has not been previously proven for metal oxides that are green-synthesized. During synthesis, the development of intermediary phases is especially noteworthy. Direct proof of the stepwise conversion mechanism is provided by the discovery of the Zn_3_(NO_3_)_2_(OH)_4_ and Cu_2_(OH)_3_NO_3_ phases.

Crucial details regarding the size and surface morphology of the NPs were also revealed by the SEM analysis. Consequently, it was determined that ZnO and CuO NPs possess a granular morphology. For every metal oxide, the morphological examination shows clear variations in the mechanisms underlying particle production. Contrary to numerous observations in the literature, ZnO and CuO NPs have consistent granular shapes and narrow size distributions. For comparison, Agarwal et al. [49] reported ZnO NPs with broader size distributions than those using other raw materials such as neem extract [11]. In this direction, we emphasize that our standardized synthesis protocol yields reasonably uniform size distributions consistent across batches, enabling reproducible nanoparticle properties under identical experimental conditions [50]. All samples showed an agglomeration tendency, which is typical of high-surface-energy nanoparticles but less evident compared to that seen in chemical production techniques. Significant aggregation was noted by Keabadile et al. [51] in chemically produced CuO NPs, necessitating the use of extra stabilizers. The remaining organic molecules in our green-synthesized particles naturally stabilize them, lowering agglomeration without sacrificing crystallinity.

FTIR spectroscopy revealed organic functional groups such as C=O, C=C, and O-H groups on NP surfaces before calcination, suggesting the presence of phenolic and flavonoid compounds that served as stabilizing and reducing agents during synthesis. Post-calcination peaks for Zn-O and Cu-O bonds evidenced the creation of pure metal oxides. Indirect but convincing evidence for a potentially unique nanoparticle generation mechanism in our system is provided by the FTIR and GC-MS results. In particular, the various and complementary chemical components of orange peel extract, such as fatty acids, terpenoids, polymethoxyflavones, and derivatized sugars, seem to work together. Our synthesis mechanism most likely consists of several cooperative processes. First, flavonoids and terpenoids reduce metal ions by acting as electron donors. Their chelating qualities also aid in stabilizing the recently created metal nuclei. Fatty acids cap the surfaces of the nanoparticles to stop them from aggregating, whereas sugars and amino acids seem to control particle shape during crystal formation. Compared to traditional green synthesis techniques, which usually rely on single reduction mechanisms, this multi-pathway approach represents a significant advancement. With its many functional groups, orange peel extract’s complex phytochemical makeup produces a special environment that boosts stability, crystallinity, and achieves appropriate size uniformity. Although additional mechanistic research (such as in situ spectroscopy) would offer conclusive evidence, our findings unequivocally show that this multi-component, ecologically safe approach has clear benefits for the creation of nanoparticles. This finding creates new opportunities for creating more eco-friendly and effective synthesis processes that draw inspiration from the intricacy of nature.

The positive zeta potential values found in all NPs indicate a positively charged surface, which enhances electrostatic contact with negatively charged bacterial cell membranes, thereby boosting antibiotic activity [52]. The zeta potential degree implies moderate colloidal stability, emphasizing that the NPs may require stabilizers for extended storage or use in certain conditions, as aggregation could diminish their effectiveness in biological applications. Furthermore, TEM examination revealed the crystalline nature of the NPs, showing that the ZnO and CuO NPs samples consist of well-defined crystal structures. The granular morphology of ZnO and CuO, along with the one seen in TEM pictures, are consistent with SEM results. High-resolution TEM scans revealed that these NPs are crystalline with well-defined crystal structures, which were produced during green synthesis when the orange peel extract induced reduction and stabilization. The close agreement between particle sizes measured by TEM/SEM and crystallite sizes calculated from XRD confirms that these NPs are predominantly monocrystalline, meaning each particle consists of a single crystal domain rather than multiple crystallites. These properties are essential for use in biological environments since it is well-known that stability can affect cell uptake and functionality. The consistent particle shapes and sizes suggest that the green synthesis method provides a reliable and repeatable way to obtain NPs with a high degree of structural uniformity, necessary for medical applications where particle shape and size can influence cell interaction.

In the field of nanoparticle-based therapeutic approaches, our investigation related to the green-synthesized ZnO and CuO NPs revealed distinct patterns of cellular interaction and biocompatibility. While each nanoparticle type demonstrates unique characteristics, their potential applications in tissue engineering and wound healing vary significantly based on their cellular response profiles. As reported by Sanaeimehr et al. [53], ZnO NPs exhibit a concentration-dependent cytotoxicity pattern. Hence, for concentrations higher than 25 mg/mL, a significant cellular stress is triggered in both L929 murine fibroblasts and primary human dermal fibroblast BJ cells. However, when examining their interaction with MG-63 osteoblast-like cells, a more positive response occurs (regarding cell viability), with lower concentrations (6.25–25 mg/mL) maintaining acceptable biocompatibility. This differential response pattern suggests potential applications in bone tissue engineering, conditioned by the careful consideration of concentration control. Nevertheless, their pronounced cytotoxicity towards fibroblasts imposes certain limitations on wound healing applications. At the same time, their inherent antimicrobial properties might still be advantageous when incorporated into specialized delivery systems. The smallest concentration could be applied for wound healing applications and has been shown in many other studies, as the one reported by Saranya et al. [54].

Continuing with CuO NPs, the study has shown a more severe cytotoxic profile across all evaluated cell lines, which aligns with current literature on metal oxide NPs behavior. The cytotoxicity evaluation of CuO nanoparticles revealed significant dose-dependent toxic effects across multiple cell lines, which aligns with current literature on metal oxide nanoparticle behavior. The dose-dependent cytotoxicity observed in L929 murine fibroblasts, BJ human dermal fibroblasts, and MG-63 osteoblast-like cells is consistent with previous studies. Ahamed et al. [55] reported similar dose-dependent cytotoxicity in human lung epithelial cells, with cell viability decreasing from 83% to 28% when exposed to 2–50 µg/mL concentrations. Our findings showing 40% viability reduction at 6.25 mg/mL and substantial cytotoxicity above 25 mg/mL follow similar patterns, though at notably higher concentrations than typically reported [56]. The severe cytotoxic effects across all three cell lines corroborate findings from multiple research groups.

Possible correlation with nanoparticle uptake efficiency is suggested by the observed dose-dependent cytotoxicity profiles, especially the increased sensitivity of fibroblast cells (L929, BJ) in comparison to osteoblast-like cells (MG-63). It is commonly known that positively charged nanoparticles, like the ZnO and CuO NPs synthesized here (ζ = +13.57 and +18.64 mV, respectively), strongly attract the negatively charged phospholipid membranes of mammalian cells electrostatically, promoting adhesion and internalization mainly through endocytic pathways [57,58]. One of the main mechanisms causing the metabolic disruption and cell death measured by our MTT assays is the subsequent intracellular release of metal ions (Zn^2+^, Cu^2+^) and/or the production of reactive oxygen species (ROS) within endo-lysosomal compartments [52,59]. Therefore, variations in absorption rates, membrane composition, and intrinsic antioxidant potential may be the cause of the heterogeneity in cytotoxic response among cell lines. This is strongly supported by studies on similarly synthesized ZnO NPs. Jo et al. [60], for example, showed that the cytotoxicity of ZnO NPs in human lung cells was directly proportional to their cellular absorption, which was impacted by the dispersant utilized. They demonstrated quantitatively that increased cell mortality and membrane damage (LDH release) were associated with higher intracellular zinc levels (as determined by ICP-AES). They also verified that the absorption was an energetically dependent and active process.

A more recent study performed by Mittag et al. [61] further supports this, demonstrating that ZnO NPs (less than 50 nm and less than 100 nm) are internalized by human intestinal cells (LT97 and Caco-2), resulting in dose-dependent cytotoxicity, apoptosis, and genotoxicity. Following NP exposure, their ICP-MS data verified elevated intracellular zinc levels, and TEM imaging showed NP accumulation in endosomes and morphological indicators of cellular stress. Furthermore, the increasingly affected LT97 cells demonstrated increased susceptibility to ZnO NP-induced disruption of the cell cycle and development of micronuclei, underscoring the significance of uptake and response mechanisms particular to cell types. The toxicological profile of metal oxide nanoparticles is largely determined by the effectiveness of NP absorption and the ion release that follows. These results support this theory. In addition, the latest work of Velumani et al. [62] on biologically produced CuO NPs in root cells of Allium cepa showed cytotoxic and genotoxic effects associated with absorption and intracellular contact with cellular structures and chromosomes. This study supports the idea that cellular internalization of CuO NPs is correlated with cellular injury and death mechanisms, even though it was carried out in plant and non-mammalian cells. The significance of NP absorption and intracellular destiny in determining cytotoxic consequences is further supported by such multi-model studies. Although we did not measure cellular internalization directly in this study, our dose-dependent cytotoxicity profiles and positive ζ-potential are consistent with uptake-mediated effects; previous work that measured intracellular metal and visualized NP internalization along with cytotoxicity supports this interpretation.

Karlsson et al. [56] demonstrated that CuO NPs exhibit higher toxicity compared to other metal oxide nanoparticles, while Wang et al. [63] showed similar cellular uptake and genotoxicity in human epithelial cells. The broad-spectrum cytotoxicity affecting both murine and human cell lines suggests universal toxic effects across species. The morphological alterations and widespread cell death observed in MG-63 cells support previous findings of metal oxide nanoparticle-induced structural changes. AshaRani et al. [64] documented similar morphological changes, including cell rounding, nuclear condensation, and apoptotic body formation. These changes indicate cellular stress and activation of multiple cell death pathways, consistent with our comprehensive cytotoxic profile. While CuO NPs demonstrate potent biological activity, their severe cytotoxic profile across multiple cell lines, particularly fibroblasts essential for wound healing, significantly limits therapeutic potential. Future research should focus on strategies to mitigate cytotoxicity while preserving antimicrobial properties, including lower concentration studies, particle size optimization, and surface modification approaches. Thus, we could sum up that both ZnO and CuO NPs might be better used in specific applications where their stronger biological effects can be controlled. To summarize, these results indicate not only the different degrees of biocompatibility among metallic oxide NPs, but also the necessity of cell-type-specific responses in defining their prospective applications. As a result, future research approaches may focus on designing complex delivery systems for ZnO and CuO NPs to exploit their positive features while minimizing cytotoxicity [65].

The antimicrobial activity of ZnO and CuO NPs is associated with their specific physicochemical features, such as nanoscale size, surface charge, and crystalline structures, which have a substantial impact on their interactions with microbial cells. According to numerous reports in the literature for comparable metal oxide nanoparticles, ZnO and CuO NPs antibacterial activity may be partially attributed to the production of reactive oxygen species (ROS) [66,67,68,69]. The reported antibacterial and dose-dependent cytotoxicity effects are compatible with ROS-mediated processes, despite not being directly assessed here. Membrane disruption through electrostatic interactions and metal ion release are other contributing causes. These include hydroxyl radicals (^•^OH), superoxide anions (O_2_^−^), and hydrogen peroxide (H_2_O_2_), which are produced when the NPs interact with biological media or are exposed to light. As mentioned by Dwivedi et al. [42], ROS inflicts oxidative stress on microbial cells, leading to damage in critical components such as lipids, proteins, and DNA. This oxidative damage impairs the structural integrity of microbial cell membranes, resulting in cell death. As a result, the ability of ZnO and CuO NPs to generate ROS is essential to their antimicrobial activity, especially against *Gram-positive* and *Gram-negative* bacteria, as well as fungi such as *C. albicans*. Another mechanism of antimicrobial action is the disintegration of microbial membranes. The positively charged surfaces of ZnO and CuO NPs interact electrostatically with negatively charged microbial membranes, resulting in enhanced permeability and leakage of vital cellular components. As mentioned by Gudkov et al. [70], this membrane disruption is more prominent in ZnO and CuO NPs due to their smaller size and stronger surface charge. Additionally, Jiang et al. [71] explain the release of metal ions, which include Zn^2+^ and Cu^2+^, that is able to enhance antibacterial action by attaching to thiol groups in proteins and affecting enzymatic activities. The small size and quasi-spherical forms of the NPs allow them to penetrate bacterial biofilms and cell membranes.

Differences in the crystalline structures and surface chemistry of NPs can explain the observed varied antimicrobial effects across microbial strains. The ZnO hexagonal wurtzite structure and CuO monoclinic shape both increase ROS formation and metal ion release. Microbial strains are susceptible to these NPs in varying degrees, with *Gram-positive* bacteria being more sensitive than *Gram-negative* bacteria and fungi. This variation is due to differences in their cell wall structures. Recent studies, such as the one performed by Hetta et al. [72], mentioned that *Gram-positive* bacteria possess a thick peptidoglycan layer but lack the additional outer membrane found in *Gram-negative* bacteria. They become more vulnerable to NP penetration and subsequent ROS-induced damage. As a result, ZnO and CuO NPs easily bypass these obstacles due to their small dimensions, high positive zeta potential, and ability to create ROS. Additionally, Lima et al. [73] indicated that *C. albicans*’ susceptibility to ZnO and CuO NPs might have been related to the fungal cell wall composition, which contains chitin and β-glucans that are more vulnerable to oxidative stress and membrane disruption.

The addition of orange peel extract in the green synthesis of these NPs significantly improves their antimicrobial activity. The inclusion of functional groups from the orange peel, such as phenolic and flavonoid compounds, stabilizes the NPs while also increasing their biological activity. Before calcination, these organic groups provide additional bioactive characteristics that contribute to antimicrobial activity. After calcination, the elimination of organic residues yields pure metal oxides, which improves the antimicrobial activity of ZnO and CuO NPs. The MBEC evaluation revealed significant differential effectiveness of these NPs, which might be attributable to numerous interrelated pathways. The green-synthesized ZnO and CuO NPs are significant inhibitors of biofilm formation, as evidenced by the measured MBEC values, which exhibit a good association with MIC results. The small, positively charged nanoparticles most likely cause the disruption of extracellular polymeric substances (EPSs) and direct harm to embedded microbiological cells by penetrating the biofilm matrix. This suggested process aligns with a wealth of research on metal oxide nanoparticles, where visible confirmation of EPS breakdown, loss of biofilm structural integrity, and bacterial cell lysis has been obtained using methods including CLSM and SEM [74,75,76,77,78]. For example, the study performed by El-Sawaf et al. [77] shows that green-synthesized CuO and ZnO NPs exhibit strong antimicrobial and antibiofilm activities against multidrug-resistant pathogens, including *Escherichia coli* and *Staphylococcus aureus*. Characterization by microscopy (SEM, TEM) confirms a small particle size (~7 nm) with the positive surface charge (zeta potential +21.5 mV), supporting the effective interaction with the negatively charged biofilm matrix. Thus, the quantitative effectiveness shown by the MBEC assay, along with the known structure–activity relationship of nanoparticles of this size and surface charge, provides strong evidence for their anti-biofilm potential, even though such direct visualization was not carried out in this study. These microscopic investigations will be given priority in future research in order to visually validate the disruptive effects measured within the study. It could be concluded that ZnO and CuO NPs are more efficient against *Gram-positive* bacteria due to their smaller size and stronger contact with the peptidoglycan layer, resulting in increased biofilm breakdown. These results underscore the importance of considering NP properties, such as size, crystalline structure, and surface chemistry, in developing effective antimicrobial treatments.

Previous studies have shown that ROS formation by similar metal oxide NPs under similar synthesis and environmental conditions [79,80,81] serve as strong support for the antimicrobial efficacy of the synthesized NPs, even though preliminary measurements of ROS generation by these NPs were not conducted in this study. For instance, ZnO and CuO NPs are known to produce ROS by redox cycling or photocatalysis (as reported by Zhang et al. [82]), which causes oxidative stress in microbial cells, resulting in membrane damage and cell death [83]. In our study, the superior antibacterial activity of ZnO and CuO NPs—particularly against *Gram-positive* bacteria (e.g., MIC = 0.31 μg/μL for *S. aureus*)—aligns with their well-documented ROS-generating capacity. The dose-dependent cytotoxicity of these NPs in fibroblast and osteoblast cells (Figure 11 and Figure 12) further supports oxidative stress as a plausible mechanism, consistent with the literature on ROS-induced cellular damage [41,83].

In addition, the NPs were thoroughly characterized, revealing several important structure–property connections that account for their varying biological performances. The larger crystallites (ZnO ~ 22 nm) displayed dose-dependent cytotoxicity. This indicates that crystallite size, as determined by XRD analysis, has a direct correlation with biocompatibility. The reason for this effect could be the result of smaller particles that have a larger surface area-to-volume ratio, which promotes deeper interactions between cells. In addition, antibacterial efficacy was significantly influenced by the crystal structure. Due to the increased reactive crystal facets, the hexagonal wurtzite structure of ZnO and the monoclinic phase of CuO produced high ROS levels, which explains their superior antibacterial efficacy (Figure 13, Figure 14 and Figure 15). Zeta potential measurements of surface charge revealed a clear relationship with microbial membrane interaction; the strongest antibacterial activity against Gram-positive bacteria (MIC = 0.31 μg/μL for *S. aureus*) was associated with the highest positive charge (CuO: +18.64 mV). Finally, the ability of biofilms to penetrate was impacted by morphological variations seen in SEM/TEM investigations (Figure 5 and Figure 8). ZnO and CuO NPs show superior activity against planktonic bacteria. These relationships between structure and property offer crucial guidelines for customizing nanoparticles for biomedical purposes. Meanwhile, further investigation into their potential applications across a broader range of tissue engineering and wound healing applications must be carried out. This comprehensive understanding of nanoparticle–cell interactions provides valuable insights for developing next-generation biomaterials, ultimately contributing to more effective tissue engineering and wound healing strategies. Moreover, these findings emphasize the need for careful consideration of both concentration and cell-specific responses when designing nanoparticle-based therapeutic approaches.

The presented work fills important gaps in methodological standardization and comparative analysis, advancing the field of green-synthesized metal oxide NPs. In this direction, we have developed ZnO and CuO NPs using a consistent orange peel extract-based protocol, in contrast to previous works that concentrate on individual NPs synthesized under different conditions (e.g., Thi et al. [31] for ZnO; Murugan et al. [13] for CuO). This allows for a direct comparison of their physicochemical and biological properties under comparable experimental conditions. Our method exposes previously unknown trends, including the higher antibacterial activity of smaller, monocrystalline NPs (e.g., CuO MIC = 0.31 μg/μL for *S. aureus*) and the inverse association between crystallite size (CuO < ZnO) and biocompatibility. Additionally, we uncover a molecular insight that is often missed in traditional single-extract studies: the synergistic function of orange peel phytochemicals (such as polymethoxyflavones and terpenoids) in mediating NP synthesis and stability.

### Limitations and Future Perspectives

Even though the physicochemical and biological characteristics of our green-synthesized NPs were not directly compared to those of chemically synthesized NPs (using identifiable precursors without orange peel extract), they could be thoroughly evaluated by comparing them with the results of well-established literature on conventional methods. Compared to those synthesized via a chemical sol-gel technique (~20–40 nm as reported by Dörner et al. [84]), the CuO NPs produced using our green method (12.65 ± 1.24 nm) had substantially smaller primary crystallite sizes. According to Bekele et al. [85] and Agarwal et al. [49], conventional chemical precipitation frequently produces broader aggregates (30–40 nm), whereas our ZnO NPs (17.47 ± 3.76 nm) show a narrower size distribution and superior morphological uniformity. Using a leaf extract from *Mimusops elengi*, Jana et al. [86] compared ZnO NPs produced by chemical precipitation against green chemistry. The average crystallite size for chemical synthesis was 32.59 nm, whereas green synthesis produced crystallites that were 32.12 nm, indicating similar sizes with better morphological regularity. In contrast to chemical approaches, the study highlighted that green synthesis provided more homogeneous surface morphology with extra adsorptive sites. In comparison to chemically manufactured CuO NPs, Sabeena et al. [87] revealed that green-produced CuO NPs exhibited greater antibacterial activity against both Gram-positive (*Bacillus subtilis*, *Pseudomonas aeruginosa*) and Gram-negative (*Escherichia coli*, *Staphylococcus aureus*, *Enterobacter*) bacteria. According to their findings, green CuO NPs showed increased bioactivity in a variety of biological evaluations, such as anti-inflammatory and antidiabetic effects. One of the main benefits of using the green synthesis technique is the improved control over size and shape, which is caused by the capping and stabilizing action of the phytochemicals found in orange peel. Our study’s dose-dependent biocompatibility profile and improved antimicrobial efficacy (e.g., MIC = 0.313 μg/μL for *S. aureus*) are consistent with earlier findings that green-synthesized metal oxide nanoparticles frequently perform better biologically than their chemically synthesized counterparts [85].

Although we did not perform pilot-scale or industrial-level synthesis, the green synthesis method relies on simple equipment (magnetic stirring, ultrasonic bath, and calcination oven) and inexpensive, widely available materials (orange peel and metal nitrates). These aspects suggest potential scalability, which merits further dedicated studies to optimize process parameters and energy consumption to assess economic and environmental viability at larger production volumes [88]. Furthermore, this study’s evaluation of NP colloidal stability solely in ultrapure water is one of its limitations. The behavior of ZnO and CuO NPs in complex biological conditions (such as DMEM with serum) is probably influenced by the creation and aggregation of protein corona, which can drastically change the particles’ hydrodynamic size, surface characteristics, and settling rate. The interpretation of the dose-dependent responses shown here depends critically on this dynamic process, which may affect the effective dose given to bacteria and cells over time. A thorough temporal DLS analysis will be incorporated into future research to establish a direct correlation between stability in biological medium and the noted cytotoxic and antibacterial effects.

In addition, even though the antimicrobial efficacy of ZnO and CuO NPs is often attributed to ROS generation in the literature [42,82,83], we note that direct experimental evidence of ROS production was not conducted in this study. Therefore, although ROS-mediated oxidative stress is still a likely mechanism, the observed effects may also be greatly influenced by additional elements such as membrane rupture, ion release, and electrostatic interactions. The green-synthesized ZnO and CuO NPs showed notable antimicrobial activity, but their dose-dependent cytotoxicity, especially at concentrations higher than 25 µg/mL, still makes direct biological use difficult. The main causes of this cytotoxicity are processes that cause oxidative stress in mammalian cells, such as ROS production and metal ion release (Zn^2+^, Cu^2+^). Future approaches will concentrate on immobilizing NPs within biocomposite scaffolds, like silk fibroin–chitosan matrices, which can regulate ion release kinetics and lessen direct cellular exposure, in order to minimize these effects and maximize their antibacterial potential. As mentioned in an earlier study, this strategy is consistent with our current investigation into functionalized scaffolds for tissue engineering and wound healing applications [89].

The study’s main finding is that the green-synthesized ZnO and CuO NPs have strong antibacterial action against common strains of clinically significant bacteria, including *S. aureus* and *E. coli* [90]. Interestingly, CuO NPs showed remarkable anti-*S. aureus* activity (MIC = 0.313 μg/μL). Although standard laboratory strains were used in this study to set a baseline for the antimicrobial properties of the NPs, the main mechanisms of action (the ions release, the formation of ROS, and membrane disruption via electrostatic interactions facilitated by the positive zeta potential) are known to be effective against bacteria despite their drug-resistance mechanisms [69,70,71,91]. Furthermore, as reported by Vindhya et al. [92], Gram-positive bacteria, such as *S. aureus*, are especially vulnerable to these non-specific processes because they do not have the same complex outer membrane as the Gram-negative bacteria. This clearly implies that our NPs may be effective against strains of these infections that are resistant to drugs, including methicillin-resistant *S. aureus* (MRSA). Because of its effective efflux pump systems and robust outer membrane, *P. aeruginosa* is known to have intrinsic resistance, which is compatible with the reported difficulty in eliminating its biofilms (high MBEC value) [93]. To directly verify the potential of these green-synthesized NPs in addressing the pressing worldwide issue of antimicrobial resistance, it would be reasonable and crucial to assess their effectiveness against a panel of clinically isolated multidrug-resistant (MDR) strains in the near future.

Moreover, the MBEC assay does not directly demonstrate the structural damage brought on by the nanoparticles, although it offers a reliable quantitative measure of biofilm suppression. Future research would benefit greatly from the use of microscopic tools like CLSM or SEM, which were not used in this study, to visualize impacts such as bacterial cell shape and aggregation, biofilm thickness decrease, and EPS matrix breakdown [74,75,76]. Strong indirect evidence for an uptake-dependent toxicity mechanism is provided by the obvious structure–activity link between NP surface charge/size and the biological response, even though the direct assessment of uptake (for example, via ICP-MS) was outside of the boundaries of this investigation. Another limitation is that we did not directly measure the cellular uptake of ZnO and CuO NPs in our experiments. While the observed cytotoxicity patterns and literature evidence strongly suggest an uptake-mediated mechanism, definitive confirmation (e.g., via TEM imaging in cells, ICP-MS quantification of intracellular ions, or uptake-inhibition assays) will be an important focus of future studies. Moreover, future studies should include direct measurement of ROS generation (e.g., via fluorescent probes) to conclusively establish the mechanistic pathways behind the antimicrobial and cytotoxic effects observed and a thorough investigation of the colloidal stability and hydrodynamic size of the nanoparticles in biologically relevant media over time to better understand the relationship between nominal dose, effective dose, and biological activity. The current study establishes phase purity, shape, and baseline biological reactions. Nevertheless, the study did not assess ion release or dissolution in biologically relevant media. Thus, the ICP-MS study of Zn^2+^ and Cu^2+^ release in DMEM ± serum and at various pH conditions, in addition to DLS and XPS, will be the main focus of future investigations. Whether the nanoparticles persist or disintegrate, as well as how any breakdown products affect biological function, will be made clear by these assays. To directly correlate environmental stability with the observed cytotoxic and antibacterial effects, future studies should include thorough temporal stability evaluations in biologically relevant media (changing pH and ionic strength).

In addition, although the calcination temperatures employed here (400 °C for ZnO and 300 °C for CuO) were effective in producing phase-pure crystalline oxides, we did not perform a systematic study of adjacent temperatures to determine their impact on crystallinity, particle size, and antimicrobial or biocompatibility outcomes. Future work will therefore extend the parameter space to establish more detailed correlations between calcination conditions and nanoparticle properties.

This study has a number of other methodological shortcomings that should be noted. To compare each treatment concentration to the negative control, we used the Student’s *t*-test; however, this method only shows which specific concentrations have statistically significant effects in comparison to untreated cells. Further statistical studies, such as trend analysis utilizing linear regression across concentration ranges or ANOVA followed by suitable post-hoc tests to directly compare across different dose levels, will be carried out as future work in order to firmly demonstrate actual dose-dependent connections.

Although our current statistical methodology is sufficient for detecting individual concentration effects, it falls short in providing complete statistical support for predictions of dose-response correlations. Furthermore, our ability to provide standardized quantitative comparisons with other studies in the area was limited because our concentration range (6.25–200 µg/mL) did not consistently allow for reliable IC50 estimations across all investigated settings and cell types. In order to properly characterize dose-response relationships and enable more rigorous IC50 values when biologically relevant, future studies should use wider concentration ranges and more thorough statistical approaches, such as non-linear regression analysis.

## 4. Materials and Methods

### 4.1. Materials

The following reagents were used for nanoparticle synthesis: cupric nitrate hemi(pentahydrate) [Cu(NO_3_)_2_·2.5H_2_O, ≥98%, Sigma-Aldrich, Darmstadt, Germany] and zinc nitrate hexahydrate [Zn(NO_3_)_2_·6H_2_O, ≥98%, Sigma-Aldrich, Darmstadt, Germany]. To prepare the orange peel extract, 2 kg of oranges were purchased from the local market. For cell viability assessment and cell culture preparation, immortalized murine fibroblast L929 cells, primary human dermal fibroblast BJ cells (both obtained from American Type Culture Collection, ATCC, Manassas, VA, USA), and MG-63 osteoblast-like cells (Cell Lines Service GmbH, Heidelberg, Germany) were maintained in Dulbecco’s Modified Eagle Medium (DMEM) supplemented with 10% fetal bovine serum (FBS) and 1% *Penicillin-Streptomycin* antibiotic solution. Cells were cultured under standard physiological conditions in a humidified atmosphere containing 5% CO_2_ at 37 °C with 90% relative humidity. The microbiological activity was performed using Nutrient Broth No. 2, Sabouraud agar, phosphate buffer saline, methanol, crystal violet (purple), and acetic acid, purchased from Sigma-Aldrich (Darmstadt, Germany). All strains tested in this study were provided by the Microorganisms Collection of the Department of Microbiology, Faculty of Biology & Research Institute of the University of Bucharest, Bucharest, Romania.

### 4.2. Preparation of Orange Peel Extract

Nearly 2 kg of fresh oranges have been purchased from the local market. The peels have been washed, cut, and dried in an oven at 40 °C for 4 h. The dried peels have been ground in a mortar for 30 min to produce a fine powder. To prepare the extract, 2 g of powder (1% *w*/*v*) in 200 mL have been introduced in deionized water and magnetically stirred at room temperature for 1 h. This procedure was followed by ultrasonic treatment at 60 °C for 1 h. The mixture was filtered through Whatman No. 1 filter paper and kept at 4 °C for future use. The pH of the resulting orange peel extract was approximately 5.2, consistent with the presence of organic acids such as citric, malic, and ascorbic acid [32,33].

### 4.3. Green Synthesis of Nanoparticles

Unless otherwise specified, all NPs were produced at a 1:1 volume ratio of orange extract to metal salt solution. ZnO and CuO NPs were synthesized in uniform environments, with variations depending on precursor composition and calcination treatment. For ZnO NPs, a 0.2 M aqueous solution of Zn(NO_3_)_2_·6H_2_O (50 mL) was mixed with 50 mL of orange peel extract and stirred for 1 h. The mixture underwent ultrasound treatment at 60 °C for 1 h, then oven-dried at 150 °C for 12 h. The resulting solid was calcined at 400 °C for 2 h with a ramp rate of 5 °C/min. The average yield was approximately 0.45 g per 100 mL batch. CuO NPs were synthesized similarly, using 50 mL of 0.2 M Cu(NO_3_)_2_·2.5H_2_O solution combined with 50 mL of orange extract. After stirring and ultrasound treatment, the mixture was dried at 150 °C and calcined at 300 °C for 2 h at a 5 °C/min heating rate. The typical yield was around 0.41 g. The product was filtered, dried at 150 °C for 12 h, and calcined at 400 °C for 2 h. The yield was about 0.48 g. The chosen calcination conditions (400 °C for ZnO, 300 °C for CuO) were selected based on preliminary optimization and our previous work, which confirmed that these temperatures yield stable crystalline oxides [48]. All synthesized nanoparticle powders were stored in airtight containers under desiccation conditions until characterization.

### 4.4. Thermogravimetric Analysis

The thermal behavior of dried powders (precursors of ZnO and CuO NPs) has been investigated using a Netzsch TG 449C STA Jupiter instrument (Netzsch, Selb, Germany) to establish the optimum calcination temperature and investigate the possible transformation of the metallic nitrates induced by the orange peel extract. The powder samples were placed in an alumina crucible and heated from room temperature to 900 °C, at a rate of 10 °C per minute, under a flow of dried air at a rate of 50 mL per minute. The evolved gases were transferred through heated transfer lines and analyzed on the fly with the help of an FTIR Tensor 27 from Bruker (Bruker Co., Ettlingen, Germany), equipped with an internal thermostatic gas cell.

### 4.5. X-Ray Diffraction (XRD) Analysis

X-ray diffraction analysis was conducted using a PANalytical Empyrean device (Malvern Panalytical, Cedar Park, TX, USA) operating at 45 kV and 40 mA in Bragg-Brentano geometry with CuKα radiation (λ = 1.5418 Å). The diffractometer featured a 0.02° Soller slit, a 1/4° fixed divergent slit, and a 1/2° anti-scatter slit on the incident beam side, along with a 0.02 mm Ni filter mounted on a PIXCel3D detector (Malvern Panalytical, Cedar Park, TX, USA) on the diffracted beam side. The measurement parameters included a scan range of 10.0000–80.0107° 2θ, a step size of 0.0263°, and a counting time per step of 255 s. Data reduction, search, and match procedures were executed using the HighScorePlus software version 3.0.e and the ICDD PDF4+ 2022 database.

### 4.6. Scanning Electron Microscopy (SEM) Analysis

To determine the size and morphology of the green-synthesized NPs, Scanning Electron Microscopy (SEM) was performed. Images were captured using a Quanta Inspect F50 (ThermoFisher Scientific, Hillsboro, OR, USA) scanning electron microscope, which is equipped with a field emission gun (FEG) providing a resolution of 1.2 nm.

### 4.7. Fourier-Transform Infrared (FTIR) Spectroscopy Analysis

The presence of functional groups within the synthesized NPs was identified using a Nicolet iS50 FTIR spectrometer (Thermo Fisher Scientific, Waltham, MA, USA), which is equipped with a DTGS detector providing high sensitivity in the range from 4000 cm^−1^ to 400 cm^−1^, and a resolution of 4 cm^−1^ by averaging 32 scans to enhance spectral quality. The measurements were conducted at room temperature. Data recording and analysis were performed using Omnic32 software.

### 4.8. GC-MS Analysis

The chemical compound profile of the obtained orange peel extract was determined using a Thermo Scientific TRACE 1310 gas chromatograph (Thermo Fisher Scientific, Waltham, MA, USA), a TSQ-8000EV0 triple quadrupole mass spectrometer (Thermo Fisher Scientific, Waltham, MA, USA), and a TriPlus RSH autosampler (Thermo Fisher Scientific, Waltham, MA, USA). The extraction of volatile compounds was performed using a Zebron ZB-5MS capillary column (30 m × 0.25 mm ID × 0.25 µm film thickness, Phenomenex, Torrance, CA, USA), using high-purity helium (99.999%) as the carrier gas and a constant flow rate of 1.0 mL/min. The electron ionization (EI) mode at 70 eV was used, with data gathering ranging from 40 to 650 m/z at 0.2 s for each scan. The injector was kept at 280 °C, and 1 µL of the sample was added with a 10:1 split ratio.

The program of the oven temperature began at 60 °C (held for 1 min), then raised at a rate of 10 °C/min to 190 °C, followed by 15 °C/min to a final operating temperature of 300 °C, which was held constant for 30 min. The transfer line and ion source temperatures were set at 280 °C and 230 °C, respectively.

To isolate non-polar compounds, 72 mL of orange peel extract was extracted twice with 35 mL portions of hexane. The combined organic layer was dried using anhydrous sodium sulfate, then concentrated to ~1 mL with a rotary evaporator (Büchi R-300, 40 °C, 300 mbar, BÜCHI Labortechnik AG, Flawil, Switzerland). A gentle stream of nitrogen was used to fully remove residual solvent, and the dried residue was re-dissolved in 1 mL of HPLC-grade ethyl acetate prior to GC-MS injection. For the analysis of polar constituents, 0.5 mL of the aqueous phase was dried under nitrogen and derivatized using 100 μL of BSTFA with 1% TMCS at 70 °C for 30 min. Chromeleon 7.3. software was used for data collection and analysis. Compound identification was accomplished by comparing mass spectra to records in the NIST library, with retention indices utilized for extra confirmation as necessary. Major components, which include terpenoids and methoxyflavones, were semi-quantified using peak regions in total ion chromatograms.

### 4.9. Zeta Potential Analysis

A DelsaMax Pro device from Beckman Coulter (Brea, CA, USA), equipped with a 532 nm laser, was utilized to measure the zeta potential. For sample preparation, the powder samples were dispersed in ultrapure water using sonication (10 min), and the resulting suspensions were injected into the equipment’s measurement cell. Six individual acquisitions were recorded for each measurement.

### 4.10. Transmission Electron Microscopy (TEM) Analysis

The transmission electron microscopy (TEM) analysis was conducted using high-resolution Titan Themis equipment from ThermoFisher Scientific (Hillsboro, OR, USA). Before performing the analysis, a small amount of each powder sample was dispersed in deionized water using ultrasonic treatment for 10 min. Subsequently, 10 μL of the solution was placed onto a 400-mesh lacey carbon-coated copper grid and allowed to dry at room temperature. The microscope was operated at an accelerating voltage of 200 kV.

### 4.11. Biological Analysis

#### 4.11.1. Cell Culture Preparation

MG-63 osteoblast-like cells (Cytone, Heidelberg, Germany) and L929 fibroblast cells (ATCC, Manassas, VA, USA) were cultured in Dulbecco’s Modified Eagle Medium (DMEM) supplemented with 10% fetal bovine serum and 1% Penicillin-Streptomycin, in standard conditions of temperature and humidity (37 °C, 5% CO_2_, 90% humidity).

For experimental procedures, cells were seeded at a density of 5 × 10^4^ cells/mL (equivalent to 5000 cells in 100 µL per well) in 96-well tissue culture plates. The plates were then incubated under standard physiological conditions for 24 h to facilitate cellular adhesion. Following the initial incubation period, the culture medium was removed and replaced with a fresh medium containing various concentrations of NPs ranging from 6.25 to 200 µg/mL, prepared through serial binary dilutions. The treated cells were subsequently incubated under standard physiological conditions for 4 days, after which cell viability assessments were performed to evaluate the biological response to nanoparticle exposure. All experiments were conducted by standard cell culture protocols, and appropriate controls were maintained throughout the study period. The experiments were performed in triplicate, *n* = 3 and statistical analysis was performed using Student *t*-test, where * *p* < 0.05, ** *p* ≤ 0.01, and *** *p* ≤ 0.001.

The morphology of the cells was assessed at 4 days using optical microscopy, with no prior sample preparation.

#### 4.11.2. Cell Viability Assessment

Cell viability assessments were conducted using the MTT (3-(4,5-dimethylthiazol-2-yl)-2,5-diphenyltetrazolium bromide) colorimetric assay. Post-incubation, the supernatant was removed, and the cell monolayer was exposed to an MTT solution in a complete culture medium, prepared by diluting the MTT stock solution (5 mg/mL in PBS) to a final concentration of 0.5 mg/mL in complete DMEM. The cells were then incubated for 2 h under standard physiological conditions (37 °C, 5% CO_2_). The principle of this method relies on the enzymatic reduction in the tetrazolium salt to formazan crystals by mitochondrial dehydrogenases present in metabolically active cells. The intensity of this conversion is directly proportional to the number of viable cells. Following the specified incubation period, the MTT-containing medium was removed, and the formazan precipitate was solubilized in dimethyl sulfoxide (DMSO). Formazan quantification was performed by measuring the optical density at λ = 570 nm using UV-VIS spectrophotometry. Relative cell viability was expressed as a percentage of the absorbance of treated samples relative to the negative control (considered 100% viability).

#### 4.11.3. Qualitative Evaluation of Antimicrobial Activity

The qualitative antimicrobial activity was performed using an adapted spot diffusion method, according to the Clinical Laboratory Standards Institute [94,95,96]. The microbial suspensions corresponding to 1.5 × 10^8^ CFU/mL were prepared from 24 h cultures on a specific medium with agar. A stock of NPs (100 μg/μL) suspension prepared with sterile physiological buffer saline (PBS) was used. Petri plates with the medium were seeded with inocula, and 10 µL of each sample was spotted. After diffusion, the dishes were incubated at 37 °C for 24 h.

#### 4.11.4. Quantitative Evaluation of Antimicrobial Activity

The minimum inhibitory concentration (MIC) assay used an adapted binary serial microdilution standard assessment in NB medium [94,95]. In a 96-well plate, for each NP sample, serial two-fold microdilutions were performed in 150 µL of broth medium seeded with the standard inoculum. The plates were incubated at 37 °C for 24 h. Visual and spectrophotometric analyses determined the MIC values by measuring the absorbance at 620 nm using the BIOTEK SYNERGY-HTX ELISA multi-mode reader (Agilent Technologies, Winooski, VT, USA) [94,95].

#### 4.11.5. Semiquantitative Assessment of Microbial Adherence to the Inert Substratum

The biofilm development on the inert substratum was determined using the same serial two-fold microdilution method [94,95]. After 24 h of incubation and MIC measurements, the medium from the plates was removed, the walls were washed three times with PBS, and the bacterial cells adhered to the walls were fixed with methanol and tinted with 1% crystal purple. The dyed biofilm was resuspended with 33% acetic acid, and the absorbance was measured at 490 nm [94,95].

### 4.12. Statistical Analysis

The data results were statistically analyzed using GraphPad Prism, version 10.4, from GraphPad Software (San Diego, CA, USA). All experiments were performed in three independent determinations. The results are expressed as ± SD (standard deviation) and analyzed using a one-way analysis of variance (one-way ANOVA) followed by a multiple comparisons assay according to the experimental method. The differences between groups/samples were considered statistically significant when the *p*-value was <0.05.

## 5. Conclusions

This study’s standardized green synthesis protocol not only complies with environmentally friendly guidelines, but also makes it possible to conduct a thorough comparison of ZnO and CuO NPs, which is a major improvement over traditional single-nanoparticle investigations. Our work lays the groundwork for future studies in nanoparticle design for tissue engineering and antimicrobial treatments by establishing a physicochemical property–biological performance correlation.

In addition, the study successfully synthesized metal oxide NPs in an environmentally friendly manner, utilizing orange peel extract as a natural reducing and capping agent. The standardized green synthesis approach used in this study allowed for direct comparative evaluation of two metal oxide NPs under identical conditions, which is a considerable step forward from previous research that focused on individual NPs. The zeta potential measurements indicate moderate colloidal stability across all samples (+11 mV to +18 mV, Section 2.6), which is typical for metal oxide nanoparticles synthesized without additional stabilizers. While the presence of residual organic groups from the orange peel extract may contribute somewhat to stabilization, this effect appears limited post-calcination and is insufficient to prevent moderate agglomeration, as also observed by SEM and TEM analyses.

By assessing the obtained results, it has been demonstrated that both ZnO and CuO NPs showed strong antibacterial action, particularly against *Gram-positive* bacteria, which was attributed to their ROS-generating ability and membrane disruption mechanisms. The study’s novel integration of cytotoxicity and antimicrobial assessments (MIC/MBEC) provided a comprehensive understanding of their dual potential in biomedical applications. Given these benefits, the dose-dependent cytotoxicity of ZnO and CuO NPs emphasizes the importance of regulated delivery systems. Future research should focus on hybrid systems that combine the characteristics of these NPs, as well as kinetic investigations to enhance their therapeutic potential. This study not only provided a sustainable alternative to existing synthesis methods, but also established a standard for comparative nanomaterial evaluation, paving the way for customized designs in tissue engineering and antimicrobial medicines. The study has demonstrated a standardized green synthesis route for metal oxide NPs using orange peel extract, which might actively participate in precursor transformation (TGA/XRD) but does not confer long-term functionalization. The technique is reproducible and does not use harmful chemicals, but it still has to be improved in terms of scalability and energy efficiency. To improve sustainability, future research should investigate lower-temperature alternatives.

## Figures and Tables

**Table 1:** The bioactive compounds found in orange peel extract.

Class	Compounds	Functional Role	Effect on NP
Terpenoids	Linalool α-Terpineol Carvone Nootkatone	electron donation (reduction), chelation, antioxidant protection, and surface stabilization	facilitates metal ion reduction, controls nucleation, prevents oxidation, and minimizes agglomeration
Polymethoxyflavones	Tangeretin Nobiletin Heptamethoxyflavone	redox activity, chelation, and morphology optimization	initiates oxide formation, promotes size and shape uniformity
Fatty Acids	Palmitic Linoleic Acid Oleic Acid Stearic Acid	capping and micelle formation, provide colloidal stability, and adjust surface energy	enhances dispersion, controls particle growth and morphology
Amino Acids and Sugars	Derivatized forms	morphology optimization during the synthesis process	influences shape and size distribution

## Data Availability

The original contributions presented in this study are included in the article. Further inquiries can be directed to the corresponding authors.

## References

[1] Zhang G., Zhen C., Yang J., Wang J., Wang S., Fang Y., Shang P. (2024). Recent advances of nanoparticles on bone tissue engineering and bone cells. Nanoscale Adv..

[2] Karbowniczek J.E., Berniak K., Knapczyk-Korczak J., Williams G., Bryant J.A., Nikoi N.D., Banzhaf M., de Cogan F., Stachewicz U. (2023). Strategies of nanoparticles integration in polymer fibers to achieve antibacterial effect and enhance cell proliferation with collagen production in tissue engineering scaffolds. J. Colloid Interface Sci..

[3] Tejashwini D.M., Harini H.V., Nagaswarupa H.P., Naik R., Deshmukh V.V., Basavaraju N. (2023). An in-depth exploration of eco-friendly synthesis methods for metal oxide nanoparticles and their role in photocatalysis for industrial dye degradation. Chem. Phys. Impact.

[4] Nadaf S.J., Jadhav N.R., Naikwadi H.S., Savekar P.L., Sapkal I.D., Kambli M.M., Desai I.A. (2022). Green synthesis of gold and silver nanoparticles: Updates on research, patents, and future prospects. OpenNano.

[5] Álvarez-Chimal R., Arenas-Alatorre J.Á., Álvarez-Pérez M.A. (2024). Nanoparticle-polymer composite scaffolds for bone tissue engineering. A review. Eur. Polym. J..

[6] Upadhyay K., Tamrakar R.K., Thomas S., Kumar M. (2023). Surface functionalized nanoparticles: A boon to biomedical science. Chem.-Biol. Interact..

[7] Nour-Djihane Mazouzi K.B., Haddad A. (2015). Effect of adding magnesium oxide nanoparticles on hydroxyl methyl cellulose based films: Physico-chemical, mechanical and rheological properties. Rom. J. Mater..

[8] Tyagi P.K., Quispe C., Herrera-Bravo J., Tyagi S., Barbhai Mrunal D., Kumar M., Dablool A.S., Alghamdi S., Batiha G.E.-S., Sharifi-Rad J. (2021). Synthesis of Silver and Gold Nanoparticles: Chemical and Green Synthesis Method and Its Toxicity Evaluation against Pathogenic Bacteria Using the ToxTrak Test. J. Nanomater..

[9] Eddy D.R., Rahmawati D., Permana M.D., Takei T., Solihudin, Suryana, Noviyanti A.R., Rahayu I. (2024). A review of recent developments in green synthesis of TiO2 nanoparticles using plant extract: Synthesis, characterization and photocatalytic activity. Inorg. Chem. Commun..

[10] Meher A., Tandi A., Moharana S., Chakroborty S., Mohapatra S.S., Mondal A., Dey S., Chandra P. (2024). Silver nanoparticle for biomedical applications: A review. Hybrid Adv..

[11] Saeed Z., Pervaiz M., Ejaz A., Hussain S., Shaheen S., Shehzad B., Younas U. (2023). Garlic and ginger extracts mediated green synthesis of silver and gold nanoparticles: A review on recent advancements and prospective applications. Biocatal. Agric. Biotechnol..

[12] Canales D.A., Piñones N., Saavedra M., Loyo C., Palza H., Peponi L., Leonés A., Baier R.V., Boccaccini A.R., Grünelwald A. (2023). Fabrication and assessment of bifunctional electrospun poly(l-lactic acid) scaffolds with bioglass and zinc oxide nanoparticles for bone tissue engineering. Int. J. Biol. Macromol..

[13] Murugan B., Rahman M.Z., Fatimah I., Anita Lett J., Annaraj J., Kaus N.H.M., Al-Anber M.A., Sagadevan S. (2023). Green synthesis of CuO nanoparticles for biological applications. Inorg. Chem. Commun..

[14] Irina Elena Doicin A.-D.G., Neacsu I.A., Ene Alexandra Cătălina Bîrcă V.L., Holban A.M., Andronescu E. (2024). Spin-coating deposition of antimicrobial zno nanoparticles on cotton fabrics for medical applications. Rom. J. Mater..

[15] Nandhini S.N., Sisubalan N., Vijayan A., Karthikeyan C., Gnanaraj M., Gideon D.A.M., Jebastin T., Varaprasad K., Sadiku R. (2023). Recent advances in green synthesized nanoparticles for bactericidal and wound healing applications. Heliyon.

[16] Ghazal H., Waqar A., Yaseen F., Shahid M., Sultana M., Tariq M., Bashir M.K., Tahseen H., Raza T., Ahmad F. (2024). Role of nanoparticles in enhancing chemotherapy efficacy for cancer treatment. Next Mater..

[17] Habibzadeh F., Sadraei S.M., Mansoori R., Singh Chauhan N.P., Sargazi G. (2022). Nanomaterials supported by polymers for tissue engineering applications: A review. Heliyon.

[18] Mitchell M.J., Billingsley M.M., Haley R.M., Wechsler M.E., Peppas N.A., Langer R. (2021). Engineering precision nanoparticles for drug delivery. Nat. Rev. Drug Discov..

[19] Namakka M., Rahman M.R., Said K.A.M.B., Abdul Mannan M., Patwary A.M. (2023). A review of nanoparticle synthesis methods, classifications, applications, and characterization. Environ. Nanotechnol. Monit. Manag..

[20] Aigbe U.O., Osibote O.A. (2024). Green synthesis of metal oxide nanoparticles, and their various applications. J. Hazard. Mater. Adv..

[21] Dheyab M.A., Oladzadabbasabadi N., Aziz A.A., Khaniabadi P.M., Al-ouqaili M.T.S., Jameel M.S., Braim F.S., Mehrdel B., Ghasemlou M. (2024). Recent advances of plant-mediated metal nanoparticles: Synthesis, properties, and emerging applications for wastewater treatment. J. Environ. Chem. Eng..

[22] Paiva-Santos A.C., Herdade A.M., Guerra C., Peixoto D., Pereira-Silva M., Zeinali M., Mascarenhas-Melo F., Paranhos A., Veiga F. (2021). Plant-mediated green synthesis of metal-based nanoparticles for dermopharmaceutical and cosmetic applications. Int. J. Pharm..

[23] Tijani N.A., Hokello J., Awojobi K.O., Marnadu R., Shkir M., Ahmad Z., Afolabi A.O., Adewinbi S.A., Adebayo I.A. (2024). Recent advances in Mushroom-mediated nanoparticles: A critical review of mushroom biology, nanoparticles synthesis, types, characteristics and applications. J. Drug Deliv. Sci. Technol..

[24] Wen J., Gao F., Liu H., Wang J., Xiong T., Yi H., Zhou Y., Yu Q., Zhao S., Tang X. (2024). Metallic nanoparticles synthesized by algae: Synthetic route, action mechanism, and the environmental catalytic applications. J. Environ. Chem. Eng..

[25] Motelica L., Vasile B.-S., Ficai A., Surdu A.-V., Ficai D., Oprea O.-C., Andronescu E., Mustățea G., Ungureanu E.L., Dobre A.A. (2023). Antibacterial Activity of Zinc Oxide Nanoparticles Loaded with Essential Oils. Pharmaceutics.

[26] Maneva M., Petrov N. (1989). On the thermal decomposition of Zn(NO3)2·6H2O and its deuterated analogue. J. Therm. Anal..

[27] Ghose J., Kanungo A. (1981). Studies on the thermal decomposition of Cu(NO3)2·3 H2O. J. Therm. Anal..

[28] Harish V., Ansari M.M., Tewari D., Gaur M., Yadav A.B., García-Betancourt M.-L., Abdel-Haleem F.M., Bechelany M., Barhoum A. (2022). Nanoparticle and Nanostructure Synthesis and Controlled Growth Methods. Nanomaterials.

[29] Altammar K.A. (2023). A review on nanoparticles: Characteristics, synthesis, applications, and challenges. Front. Microbiol..

[30] Motelica L., Vasile B.-S., Ficai A., Surdu A.-V., Ficai D., Oprea O.-C., Andronescu E., Jinga D.C., Holban A.M. (2022). Influence of the Alcohols on the ZnO Synthesis and Its Properties: The Photocatalytic and Antimicrobial Activities. Pharmaceutics.

[31] Doan Thi T.U., Nguyen T.T., Thi Y.D., Ta Thi K.H., Phan B.T., Pham K.N. (2020). Green synthesis of ZnO nanoparticles using orange fruit peel extract for antibacterial activities. RSC Adv..

[32] Bratovcic A., Dautovic A. (2024). Green Synthesis of Silver Nanoparticles Using Aqueous Orange and Lemon Peel Extract and Evaluation of Their Antimicrobial Properties. Adv. Nanopart..

[33] Skiba M.I., Vorobyova V.I. (2019). Synthesis of Silver Nanoparticles Using Orange Peel Extract Prepared by Plasmochemical Extraction Method and Degradation of Methylene Blue under Solar Irradiation. Adv. Mater. Sci. Eng..

[34] Maqbool Z., Khalid W., Atiq H.T., Koraqi H., Javaid Z., Alhag S.K., Al-Shuraym L.A., Bader D.M.D., Almarzuq M., Afifi M. (2023). Citrus Waste as Source of Bioactive Compounds: Extraction and Utilization in Health and Food Industry. Molecules.

[35] Shehata M.G., Awad T.S., Asker D., El Sohaimy S.A., Abd El-Aziz N.M., Youssef M.M. (2021). Antioxidant and antimicrobial activities and UPLC-ESI-MS/MS polyphenolic profile of sweet orange peel extracts. Curr. Res. Food Sci..

[36] Matei A., Stoian M., Crăciun G., Țucureanu V. (2024). Chemical Synthesis and Characterization of Fatty Acid-Capped ZnO Nanoparticles. J. Compos. Sci..

[37] Bouttier-Figueroa D.C., Cortez-Valadez J.M., Flores-Acosta M., Robles-Zepeda R.E. (2023). Synthesis of Metallic Nanoparticles Using Plant’s Natural Extracts: Synthesis Mechanisms and Applications: Synthesis of Metallic Nanoparticles Using Plant’s Natural Extracts. Biotecnia.

[38] Bhattacharjee R., Negi A., Bhattacharya B., Dey T., Mitra P., Preetam S., Kumar L., Kar S., Das S.S., Iqbal D. (2023). Nanotheranostics to target antibiotic-resistant bacteria: Strategies and applications. OpenNano.

[39] Dananjaya S.H.S., Kumar R.S., Yang M., Nikapitiya C., Lee J., De Zoysa M. (2018). Synthesis, characterization of ZnO-chitosan nanocomposites and evaluation of its antifungal activity against pathogenic Candida albicans. Int. J. Biol. Macromol..

[40] Bhattacharjee S. (2016). DLS and zeta potential—What they are and what they are not?. J. Control. Release.

[41] Vasile O.R., Serdaru I., Andronescu E., Truşcă R., Surdu V.A., Oprea O., Ilie A., Vasile B.Ş. (2015). Influence of the size and the morphology of ZnO nanoparticles on cell viability. Comptes Rendus Chim..

[42] Dwivedi S., Wahab R., Khan F., Mishra Y.K., Musarrat J., Al-Khedhairy A.A. (2014). Reactive Oxygen Species Mediated Bacterial Biofilm Inhibition via Zinc Oxide Nanoparticles and Their Statistical Determination. PLoS ONE.

[43] Motelica L., Oprea O.-C., Vasile B.-S., Ficai A., Ficai D., Andronescu E., Holban A.M. (2023). Antibacterial Activity of Solvothermal Obtained ZnO Nanoparticles with Different Morphology and Photocatalytic Activity against a Dye Mixture: Methylene Blue, Rhodamine B and Methyl Orange. Int. J. Mol. Sci..

[44] Gold K., Slay B., Knackstedt M., Gaharwar A.K. (2018). Antimicrobial Activity of Metal and Metal-Oxide Based Nanoparticles. Adv. Ther..

[45] Ali D., Muneer I., Pirzada M., Ejaz T., Yasmeen F., Bashir F., Hanif M., Aziz M.H., Wahab R. (2025). Green-mediated sol-gel fabrication of CuO/ZnO nanoparticles from orange peel extract for environmental remediation: Insights from x-ray diffraction analysis using various models. Phys. Scr..

[46] Ananda Murthy H.C., Zeleke T.D., Tan K.B., Ghotekar S., Alam M.W., Balachandran R., Chan K.-Y., Sanaulla P.F., Anil Kumar M.R., Ravikumar C.R. (2021). Enhanced multifunctionality of CuO nanoparticles synthesized using aqueous leaf extract of Vernonia amygdalina plant. Results Chem..

[47] Abomuti M.A., Danish E.Y., Firoz A., Hasan N., Malik M.A. (2021). Green Synthesis of Zinc Oxide Nanoparticles Using Salvia officinalis Leaf Extract and Their Photocatalytic and Antifungal Activities. Biology.

[48] Denisa-Maria Radulescu I.A.N., Vasile B.S., Vasile-Adrian Surdu E.A. (2025). Green Synthesis of Copper, Zinc, and Magnesium Oxide Nanoparticles Using Orange Peel Extract. U.P.B. Sci. Bull..

[49] Agarwal H., Venkat Kumar S., Rajeshkumar S. (2017). A review on green synthesis of zinc oxide nanoparticles—An eco-friendly approach. Resour. Effic. Technol..

[50] Said Z., Pandey A.K., Tiwari A.K., Kalidasan B., Jamil F., Thakur A.K., Tyagi V.V., Sarı A., Ali H.M. (2024). Nano-enhanced phase change materials: Fundamentals and applications. Prog. Energy Combust. Sci..

[51] Keabadile O.P., Aremu A.O., Elugoke S.E., Fayemi O.E. (2020). Green and Traditional Synthesis of Copper Oxide Nanoparticles-Comparative Study. Nanomaterials.

[52] Wang L., Hu C., Shao L. (2017). The antimicrobial activity of nanoparticles: Present situation and prospects for the future. Int. J. Nanomed..

[53] Sanaeimehr Z., Javadi I., Namvar F. (2018). Antiangiogenic and antiapoptotic effects of green-synthesized zinc oxide nanoparticles using Sargassum muticum algae extraction. Cancer Nanotechnol..

[54] Saranya S., Vijayaranai K., Pavithra S., Raihana N., Kumanan K. (2017). In vitro cytotoxicity of zinc oxide, iron oxide and copper nanopowders prepared by green synthesis. Toxicol. Rep..

[55] Ahamed M., Siddiqui M.A., Akhtar M.J., Ahmad I., Pant A.B., Alhadlaq H.A. (2010). Genotoxic potential of copper oxide nanoparticles in human lung epithelial cells. Biochem. Biophys. Res. Commun..

[56] Karlsson H.L., Cronholm P., Gustafsson J., Möller L. (2008). Copper Oxide Nanoparticles Are Highly Toxic: A Comparison between Metal Oxide Nanoparticles and Carbon Nanotubes. Chem. Res. Toxicol..

[57] Fröhlich E. (2012). The role of surface charge in cellular uptake and cytotoxicity of medical nanoparticles. Int. J. Nanomed..

[58] Foroozandeh P., Aziz A.A. (2018). Insight into Cellular Uptake and Intracellular Trafficking of Nanoparticles. Nanoscale Res. Lett..

[59] Vranic S., Boggetto N., Contremoulins V., Mornet S., Reinhardt N., Marano F., Baeza-Squiban A., Boland S. (2013). Deciphering the mechanisms of cellular uptake of engineered nanoparticles by accurate evaluation of internalization using imaging flow cytometry. Part. Fibre Toxicol..

[60] Jo M.-R., Chung H.-E., Kim H.-J., Bae S.-H., Go M.-R., Yu J., Choi S.-J. (2016). Effects of zinc oxide nanoparticle dispersants on cytotoxicity and cellular uptake. Mol. Cell. Toxicol..

[61] Mittag A., Hoera C., Kämpfe A., Westermann M., Kuckelkorn J., Schneider T., Glei M. (2021). Cellular Uptake and Toxicological Effects of Differently Sized Zinc Oxide Nanoparticles in Intestinal Cells. Toxics.

[62] Velumani P., Palani N., Antalin Casmie A., Senthilvel R., Parthasarthy V. (2025). Cellular and chromosomal interaction of bio-synthesized copper oxide nanoparticles—Induced nano-cytotoxicity and genotoxicity. Toxicol. Vitr..

[63] Wang Z., Li N., Zhao J., White J.C., Qu P., Xing B. (2012). CuO Nanoparticle Interaction with Human Epithelial Cells: Cellular Uptake, Location, Export, and Genotoxicity. Chem. Res. Toxicol..

[64] AshaRani P.V., Low Kah Mun G., Hande M.P., Valiyaveettil S. (2009). Cytotoxicity and Genotoxicity of Silver Nanoparticles in Human Cells. ACS Nano.

[65] Zerboni A., Bengalli R., Baeri G., Fiandra L., Catelani T., Mantecca P. (2019). Mixture Effects of Diesel Exhaust and Metal Oxide Nanoparticles in Human Lung A549 Cells. Nanomaterials.

[66] Sirelkhatim A., Mahmud S., Seeni A., Kaus N.H.M., Ann L.C., Bakhori S.K.M., Hasan H., Mohamad D. (2015). Review on Zinc Oxide Nanoparticles: Antibacterial Activity and Toxicity Mechanism. Nano-Micro Lett..

[67] Lakshmi Prasanna V., Vijayaraghavan R. (2015). Insight into the Mechanism of Antibacterial Activity of ZnO: Surface Defects Mediated Reactive Oxygen Species Even in the Dark. Langmuir.

[68] Hwang C., Choi M.-H., Kim H.-E., Jeong S.-H., Park J.-U. (2022). Reactive oxygen species-generating hydrogel platform for enhanced antibacterial therapy. NPG Asia Mater..

[69] Applerot G., Lellouche J., Lipovsky A., Nitzan Y., Lubart R., Gedanken A., Banin E. (2012). Understanding the antibacterial mechanism of CuO nanoparticles: Revealing the route of induced oxidative stress. Small.

[70] Gudkov S.V., Burmistrov D.E., Fomina P.A., Validov S.Z., Kozlov V.A. (2024). Antibacterial Properties of Copper Oxide Nanoparticles (Review). Int. J. Mol. Sci..

[71] Jiang W., Mashayekhi H., Xing B. (2009). Bacterial toxicity comparison between nano- and micro-scaled oxide particles. Environ. Pollut..

[72] Hetta H.F., Ramadan Y.N., Al-Harbi A.I., Ahmed E.A., Battah B., Abd Ellah N.H., Zanetti S., Donadu M.G. (2023). Nanotechnology as a Promising Approach to Combat Multidrug Resistant Bacteria: A Comprehensive Review and Future Perspectives. Biomedicines.

[73] Lima S.L., Colombo A.L., de Almeida Junior J.N. (2019). Fungal Cell Wall: Emerging Antifungals and Drug Resistance. Front. Microbiol..

[74] Ahmed B., Ameen F., Rizvi A., Ali K., Sonbol H., Zaidi A., Khan M.S., Musarrat J. (2020). Destruction of Cell Topography, Morphology, Membrane, Inhibition of Respiration, Biofilm Formation, and Bioactive Molecule Production by Nanoparticles of Ag, ZnO, CuO, TiO2, and Al2O3 toward Beneficial Soil Bacteria. ACS Omega.

[75] Mountcastle S.E., Vyas N., Villapun V.M., Cox S.C., Jabbari S., Sammons R.L., Shelton R.M., Walmsley A.D., Kuehne S.A. (2021). Biofilm viability checker: An open-source tool for automated biofilm viability analysis from confocal microscopy images. npj Biofilm. Microbiomes.

[76] Mhade S., Kaushik K.S. (2023). Tools of the Trade: Image Analysis Programs for Confocal Laser-Scanning Microscopy Studies of Biofilms and Considerations for Their Use by Experimental Researchers. ACS Omega.

[77] El-Sawaf A.K., El-Moslamy S.H., Kamoun E.A., Hossain K. (2024). Green synthesis of trimetallic CuO/Ag/ZnO nanocomposite using Ziziphus spina-christi plant extract: Characterization, statistically experimental designs, and antimicrobial assessment. Sci. Rep..

[78] Lahiri D., Ray R.R., Sarkar T., Upadhye V.J., Ghosh S., Pandit S., Pati S., Edinur H.A., Abdul Kari Z., Nag M. (2022). Anti-biofilm efficacy of green-synthesized ZnO nanoparticles on oral biofilm: In vitro and in silico study. Front. Microbiol..

[79] Ghobadian M., Nabiuni M., Parivar K., Fathi M., Pazooki J. (2015). Toxic effects of magnesium oxide nanoparticles on early developmental and larval stages of zebrafish (Danio rerio). Ecotoxicol. Environ. Saf..

[80] Ramezani Farani M., Farsadrooh M., Zare I., Gholami A., Akhavan O. (2023). Green Synthesis of Magnesium Oxide Nanoparticles and Nanocomposites for Photocatalytic Antimicrobial, Antibiofilm and Antifungal Applications. Catalysts.

[81] Liang Y.-P., Chan Y.-B., Aminuzzaman M., Shahinuzzaman M., Djearamane S., Thiagarajah K., Leong S.-Y., Wong L.-S., Tey L.-H. (2025). Green Synthesis and Characterization of Copper Oxide Nanoparticles from Durian (Durio zibethinus) Husk for Environmental Applications. Catalysts.

[82] Zhang L., Jiang Y., Ding Y., Povey M., York D. (2007). Investigation into the antibacterial behaviour of suspensions of ZnO nanoparticles (ZnO nanofluids). J. Nanopart. Res..

[83] Azam A., Ahmed A.S., Oves M., Khan M.S., Memic A. (2012). Size-dependent antimicrobial properties of CuO nanoparticles against Gram-positive and -negative bacterial strains. Int. J. Nanomed..

[84] Dörner L., Cancellieri C., Rheingans B., Walter M., Kägi R., Schmutz P., Kovalenko M.V., Jeurgens L.P.H. (2019). Cost-effective sol-gel synthesis of porous CuO nanoparticle aggregates with tunable specific surface area. Sci. Rep..

[85] Bekele B., Degefa A., Tesgera F., Jule L.T., Shanmugam R., Priyanka Dwarampudi L., Nagaprasad N., Ramasamy K. (2021). Green versus Chemical Precipitation Methods of Preparing Zinc Oxide Nanoparticles and Investigation of Antimicrobial Properties. J. Nanomater..

[86] Subhadip Jana P.B., Banerjee S. (2024). Comparative Evaluation of Synthesized Zinc Oxide Nanoparticles by Chemical Precipitation and Green Chemistry in Removal of Methylene Blue. Lett. Appl. NanoBioScience.

[87] Sabeena G., Rajaduraipandian S., Pushpalakshmi E., Alhadlaq H.A., Mohand R., Annadurai G., Ahamed M. (2022). Green and chemical synthesis of CuO nanoparticles: A comparative study for several in vitro bioactivities and in vivo toxicity in zebrafish embryos. J. King Saud Univ.-Sci..

[88] Bagheri A.R., Aramesh N., Hasnain M.S., Nayak A.K., Varma R.S. (2023). Greener fabrication of metal nanoparticles using plant materials: A review. Chem. Phys. Impact.

[89] Radulescu D.-M., Andronescu E., Vasile O.R., Ficai A., Vasile B.S. (2024). Silk fibroin-based scaffolds for wound healing applications with metal oxide nanoparticles. J. Drug Deliv. Sci. Technol..

[90] El-Khawaga A.M., Elsayed M.A., Gobara M., Suliman A.A., Hashem A.H., Zaher A.A., Mohsen M., Salem S.S. (2023). Green synthesized ZnO nanoparticles by Saccharomyces cerevisiae and their antibacterial activity and photocatalytic degradation. Biomass Convers. Biorefin..

[91] SelvaKumar M., Jeyamurugan R., Jose P.A., Ponvel K.M., Sundarajan M. (2025). Green Synthesis, Characterization, and Biological Evaluation of CuO–ZnO Nanoparticles Using Cyphostemma Setosum Leaf Extract. ChemistrySelect.

[92] Vindhya P.S., Kavitha V.T. (2021). Comparative study of antibacterial activity of zinc oxide and copper oxide nanoparticles synthesized by green method. AIP Conf. Proc..

[93] Uthra C., Nagaraj K., Wadaan M.A., Karuppiah C., Maity P., Baabbad A., Kaliyaperumal R., Venkatachalapathy R., Shah F., Kumar P. (2024). Zinc and Copper Oxide Nanoparticles: Pioneering Antibacterial and Antibiofilm Strategies for Environmental Restoration against Antibiotic-Resistant Bacteria. Materials.

[94] Spoială A., Ilie C.-I., Trușcă R.-D., Oprea O.-C., Surdu V.-A., Vasile B.Ș., Ficai A., Ficai D., Andronescu E., Dițu L.-M. (2021). Zinc Oxide Nanoparticles for Water Purification. Materials.

[95] Ilie C.-I., Spoiala A., Chircov C., Dolete G., Oprea O.-C., Vasile B.-S., Crainiceanu S.A., Nicoara A.-I., Marinas I.C., Stan M.S. (2024). Antioxidant, Antitumoral, Antimicrobial, and Prebiotic Activity of Magnetite Nanoparticles Loaded with Bee Pollen/Bee Bread Extracts and 5-Fluorouracil. Antioxidants.

[96] CLSI (2021). CLSI Supplement M100—Performance Standards for Antimicrobial Susceptibility Testing.

